# Molecular endoscopy with next-generation sequencing improves diagnosis of cholangiocarcinoma in patients with extrahepatic biliary strictures

**DOI:** 10.1016/j.jhepr.2026.101788

**Published:** 2026-02-16

**Authors:** Anne-Cécile Brunac, Adrian Culetto, Hadrien Reboul, Karl Barange, Louis Buscail, Nadim Fares, Ronan Guillemin, Emily Alouani, David Grand, Solène Evrard, Céline Basset, Jean-Marie Peron, Janick Selves

**Affiliations:** 1 Department of Pathology, Institut Universitaire du Cancer de Toulouse - Oncopole, Toulouse University Hospital, 1 avenue Irène Joliot-Curie, 31059 Toulouse cedex 9, France; 2 Department of Gastroenterology and Pancreatology, Toulouse University Hospital, 1 avenue Jean Poulhès, TSA 50032, Toulouse Cedex 9, 31059, France; 3 Department of Hepatology, Toulouse University Hospital, 1 avenue Jean Poulhès, TSA 50032, Toulouse Cedex 9, 31059, France; 4 Department of Digestive Oncology, Toulouse University Hospital, 1 avenue Jean Poulhès, TSA 50032, Toulouse Cedex 9, 31059, France

**Keywords:** Extrahepatic biliary stricture, ERCP, NGS, Cholangiocarcinoma, Primary sclerosing cholangitis

## Abstract

**Background & Aims:**

Differentiating benign from malignant extrahepatic biliary strictures remains a diagnostic challenge, despite advances in sampling techniques and ancillary tests. Next-generation sequencing (NGS) may improve diagnostic performance and enable essential molecular profiling for malignancies. We aimed to assess the diagnostic performance of targeted NGS compared to cytohistology and fluorescence *in situ* hybridization (FISH), using samples exclusively collected via optimized single-operator cholangioscopy.

**Methods:**

We prospectively enrolled 104 patients with extrahepatic biliary strictures or suspected cholangiocarcinoma. A definitive diagnosis was established through cytohistology and clinical follow-up. In total, 445 samples (265 brushings and 180 biopsies) were collected. Targeted DNA sequencing was performed using a custom-designed 50-gene panel. FISH was retrospectively performed in a subgroup of 42 patients. The diagnostic performance of all three modalities was compared.

**Results:**

NGS achieved a sensitivity of 82.2% for malignancy, significantly higher than cytohistology alone (59.2%, *p* = 1.9x10^-3^). Combining both modalities increased sensitivity to 89.5% (*p* = 4.1x10^-2^). In the 42-patient subgroup, combining NGS and cytohistology achieved 97.2% sensitivity, superior to cytohistology (66.7%), FISH (81.2%), the combination of cytohistology and FISH (86.1%) and NGS alone (86.1%). Among malignant cases, NGS succeeded in 96.1%, enabling early molecular profiling for these patients.

**Conclusions:**

The combination of cytohistology and NGS significantly improves diagnostic performance for cholangiocarcinoma in patients with extrahepatic biliary strictures using specimens obtained via single-operator cholangioscopy. This approach also enables early molecular profiling of low-cellularity specimens and may reduce diagnostic delays while optimizing therapeutic decision-making.

**Impact and implications:**

Differentiating benign from malignant extrahepatic biliary strictures remains a major clinical challenge, justifying the need for more sensitive and specific diagnostic tools beyond conventional cytohistology. Our findings show that combining next-generation sequencing with cytohistology significantly improves diagnostic performance, enables early molecular profiling, and is easily applicable in clinical laboratories. These results are especially relevant for patients with suspected cholangiocarcinoma and patients with primary sclerosing cholangitis, where timely and accurate diagnosis is critical and often difficult. Integrating next-generation sequencing into routine diagnostic workflows could reduce diagnostic delays and guide therapeutic decisions in patients with biliary tract lesions.

## Highlights

• Targeted NGS improves malignancy detection in extrahepatic biliary strictures.
• NGS shows higher sensitivity than cytohistology and FISH alone.
• Combining NGS and cytohistology yields the highest diagnostic performance.
• NGS enables early molecular profiling to guide therapeutic decision-making.

## Graphical abstract

**Figure:**
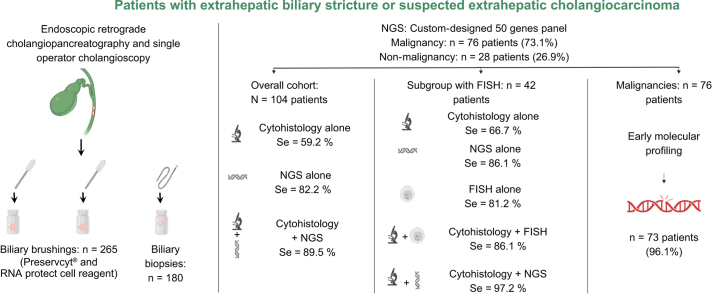

## Introduction

Extrahepatic biliary strictures (EBS) can result from both malignant and benign causes, presenting a major diagnostic challenge. One of the main benign causes of EBS is primary sclerosing cholangitis (PSC), which is associated with an increased risk of cholangiocarcinoma (CCA), with an annual risk of 1.5-2% and a 400-fold increase compared to the general population.^1^ Other benign causes of extrahepatic biliary strictures include infection-related cholangitis, IgG4-mediated cholangitis and pancreatitis, chronic pancreatitis, extrinsic compression by a pancreatic fluid collection, iatrogenic injuries, vascular conditions, and Mirizzi syndrome.^2^ Malignant strictures are most commonly due to extrahepatic CCA (eCCA), pancreatic adenocarcinoma, ampullary cancer and, less frequently, gallbladder cancer, hepatocellular carcinoma and metastatic cancers.^2^^,^^3^ EBS pose a major diagnostic challenge due to limited accessibility for tissue sampling and the potential for benign mimics, such as PSC, which can lead to diagnostic pitfalls.

Endoscopic retrograde cholangiopancreatography (ERCP) remains the gold standard technique, enabling collection of both biliary brushings and forceps biopsies. However, the sensitivity for detecting malignant biliary strictures is limited (45–56% for brushings and 48–67% for biopsies, despite excellent specificity of approximately 99%), and combining both modalities yields only a moderate increase in sensitivity.^4^^,^^5^ Although ERCP with cholangioscopy-directed biopsies has moderately improved diagnostic performance, particularly in PSC cases, and is feasible in referral hospitals,^1^ its performance remains insufficient.[6], [7], [8], [9], [10] This leads to patients frequently undergoing multiple procedures and delays in therapeutic decision-making. To improve the detection of malignant strictures, several ancillary techniques, most of them based on molecular alterations, can be used.^11^ Although FISH has been the most evaluated method to date, it is not widely used in routine practice and its sensitivity remains limited, underscoring the need to implement new technologies.

CCAs are characterized by numerous tumor-associated gene alterations, some prevalent across all anatomical sites (*KRAS* and *TP53* mutations), others more subtype-specific (*IDH1/2* mutations and *FGFR2* fusions in intrahepatic CCA [iCCA], *ERBB2* amplifications in eCCA and gallbladder adenocarcinomas). Several of these alterations represent actionable targets, positioning molecular profiling as a key step in therapeutic management.^12^^,^^13^ The utility of next-generation sequencing (NGS) for diagnosing biliary strictures has been investigated by few teams, with promising initial results.[14], [15], [16]

Building on similar approaches, the first objective of this study was to evaluate the diagnostic performance for malignancy of targeted NGS in EBS and compare it with FISH, for the first time in a prospective cohort comprising solely extrahepatic biliary samples obtained through optimized, single-operator cholangioscopy using the SpyGlass™ system. The second objective was to assess the feasibility of early molecular profiling of eCCA on both DNA and RNA.

## Patients and methods

### Study cohort

All consecutive patients diagnosed with EBS and/or suspected eCCA were prospectively enrolled in our tertiary center, Toulouse University Hospital, France, between November 2020 and June 2025. Samples were collected during procedures with standard ERCP and/or single-operator cholangioscopy (SOC) with the SpyGlass™ system. All patients were aged over 18 years and provided informed consent. Ethical approval for this study was obtained from Toulouse University Hospital (Approval number: 2023-065), with full compliance with ethical standards. All research was conducted in accordance with both the Declarations of Helsinki and Istanbul. Personal and medical data, including demographic, clinical, endoscopic, and follow-up information, were collected and processed for research purposes. A definitive diagnosis of malignancy was established based on histological or cytological evidence of at least high-grade dysplasia, clinical or radiological progression during follow-up, or malignancy-related death.

**Fig. 1:**
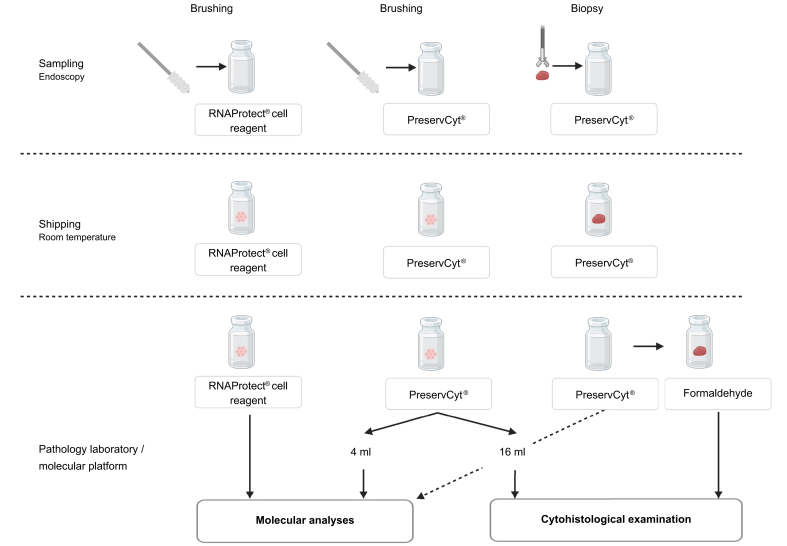
Workflow protocol. The workflow protocol was established through collaboration between endoscopists, pathologists, and the molecular biology platform for the collection and processing of the extrahepatic biliary samples.

A specific protocol was established for collecting and processing the different samples, which is detailed in the supplementary methods and summarized in Fig. 1. We compared two sampling modalities: biliary brushings and biliary biopsies, and two types of preservative solutions: PreservCyt® solution (Hologic Corp., USA), commonly used in clinical laboratories, and RNAprotect® Cell Reagent (Qiagen, Germany), which preserves nucleic acids with high quality. Briefly, all samples were collected following the dedicated protocol developed collaboratively by endoscopists, pathologists, and the molecular biology platform. Whenever possible, two brushings and at least one biopsy were obtained. Upon receipt at the pathology laboratory/molecular platform, biopsies were formalin-fixed and paraffin-embedded for cytohistological examination. Cell pellets from brushings preserved in RNAProtect® Cell Reagent were used exclusively for molecular analyses, while brushings collected in PreservCyt® were divided, with 4 ml allocated to molecular testing and 16 ml processed for cytohistological evaluation (cell blocks).

### Cytohistological examination

Slides from biliary brushings and biopsies were reviewed by two expert gastrointestinal pathologists (JS and ACB). Cytohistological findings were categorized using a simplified classification based on the WHO system:^17^

− Negative for malignancy: no morphological abnormality, reactive changes, lesions indeterminate for dysplasia, or low-grade dysplasia.
− Positive for malignancy: high-grade dysplasia, *in situ* carcinoma or invasive carcinoma.

### DNA and RNA sequencing

All specimens including brushings in PreservCyt®, brushings in RNAProtect® Cell Reagent, and PreservCyt® biopsy supernatants, were processed uniformly. When multiple samples were available for a patient, all were processed and, if qualified, sequenced. Capture-based targeted NGS was performed on an Illumina® NextSeq 550Dx in paired-end sequencing (2x149 cycles) (San Diego, CA) using a custom-designed panel targeting 50 genes for DNA sequencing, routinely utilized in our clinical laboratory for molecular profiling of solid tumors, enabling the detection of single nucleotide variants and copy number variations of relevant genes. RNA sequencing was performed using our custom-designed panel targeting 98 genes, routinely used in our clinical laboratory. Protocols of DNA and RNA sequencing and lists of the genes are described in the supplementary methods and Table S1.

Procedures were classified based on the most significant molecular alteration detected across all available samples: “Pathogenic” if at least one pathogenic or likely pathogenic variant was identified in any sample; “VUS” if only variants of unknown significance were detected, without any pathogenic variants; and “Wild type” if no such variants were found in any sample.

### Fluorescence *in situ* hybridization (FISH) analysis

Hybridization was performed on 4-μm-thick sections of biopsy specimens or cell blocks. The UroVysion® assay was employed as described previously.^18^ Results were assessed by quantifying the number of signals from centromeric probes for chromosomes CEP3 (red), CEP7 (green), CEP17 (aqua), and the locus-specific identifier 9p21 (gold) in atypical cells. Specimens were classified as “positive” if the following criteria were met: presence of >2 signals for any of CEP3, CEP7, or CEP17 in ≥4 cells (or >10%), or the loss of both locus-specific identifier 9p21 signals in ≥12 cells. A minimum of 25 well-defined, clearly visualized, and non-overlapping tumor nuclei were required for the analysis. All slides were reviewed independently by two experienced gastrointestinal pathologists (ACB and JS).

### HER2 status

HER2 status was evaluated by immunohistochemistry +/- *in situ* hybridization on FFPE blocks of CCAs according to the procedure usually used in the laboratory (detailed in the supplementary methods).

### Statistics

Baseline characteristics were reported as counts and percentages for categorical variables and as the mean with minimum and maximum values for continuous variables. Sensitivity, specificity, positive predictive value (PPV), and negative predictive value (NPV) were calculated retrospectively for each test relative to the definitive diagnosis using standard 2 × 2 contingency tables. To calculate the diagnostic performances, we considered only the first procedure performed for each patient. In cases where a patient underwent multiple procedures, only the earliest result was included in the analysis to avoid bias due to overrepresentation of patients with multiple procedures. Comparisons of sensitivity and specificity between tests were performed using the exact McNemar test, while PPV and NPV were compared as described by Moskowitz and Pepe.^19^ Statistical significance was defined as a *p-*value <0.05. All analyses were conducted using R statistical software (version 4.4).

## Results

### Cohort description

The study included 104 patients who underwent 127 procedures (15 patients with >1 procedure), all presenting with EBS and/or suspected eCCA diagnosed by MRI. Comprehensive clinical and endoscopic characteristics for the 104 patients are summarized in Table 1. Comprehensive clinical, endoscopic, sampling, pathological, therapeutic and follow-up data for each procedure are detailed in Table S2.

**Table 1:** Clinical and endoscopic characteristics for 104 patients.

Clinicopathological characteristics of all patients (N = 104)	
Gender, n (%)	
Female	34 (32.7)
Male	70 (67.3)
Mean age, years (range)	66.7 (24-87)
Repeat procedure, n (%)	
Yes	15 (14.4)
No	89 (85.6)
Endoscopic lesion, n (%)	
Stricture	78 (75)
Mass	6 (5.8)
Stricture and mass	17 (16.3)
Inflammatory	3 (2.9)
Location of lesion, n (%)	
Perihilar	50 (48.1)
Perihilar and distal	7 (6.7)
Distal	47 (45.2)
Definitive diagnosis, n (%)	
Malignant causes	76 (73.1)
Cholangiocarcinoma	55 (52.9)
Pancreatic adenocarcinoma	8 (7.7)
Ampullary adenoma with high-grade dysplasia/adenocarcinoma	3 (2.9)
Gallbladder adenocarcinoma	3 (2.9)
Intraductal papillary neoplasm of the bile duct with high-grade dysplasia	2 (1.9)
Biliary high-grade dysplasia (on primary sclerosing cholangitis)	1 (1)
Duodenal adenocarcinoma	1 (1)
Hepatocellular carcinoma	1 (1)
Neuroendocrine carcinoma of the main bile duct	1 (1)
Extrinsic compression by carcinomatosis	1 (1)
Non-malignant causes	28 (26.9)
Primary sclerosing cholangitis/overlap syndrome	11 (10.6)
Benign stricture (infectious, iatrogenic, post-acute pancreatitis, post-traumatic)	10 (9.6)
Lithiasis	3 (2.9)
IgG4-related cholangitis	2 (1.9)
Secondary biliary cirrhosis	1 (1)
Mirizzi syndrome	1 (1)

In total, 84 patients (80.8%) presented clinical symptoms (including jaundice, abdominal pain, pruritus, alteration of general health condition, fever, nausea), and five patients (4.8%) presented cholestasis without symptoms. A total of 11 patients (10.6%) were included in the context of a PSC follow-up. The mean follow-up duration was 14.8 months (95% CI 12.3-17.2).

**Fig. 2:**
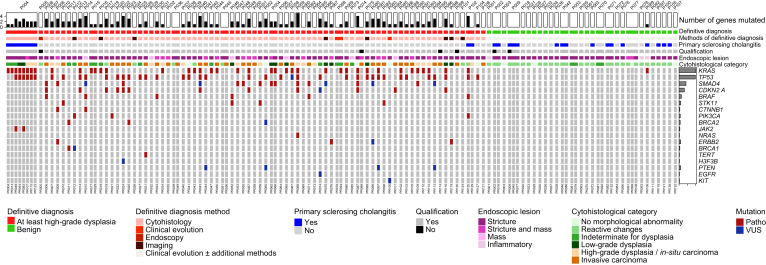
Heatmap illustrating the mutational landscape of the 104 patients included in the study. Each column corresponds to a single ERCP procedure (procedure identifiers shown at the bottom), ordered according to the definitive diagnosis, and columns are grouped by patient (patient identifiers shown at the top). The heatmap displays detected pathogenic variants (dark red) and variants of unknown significance (dark blue) across the targeted genes. The bar plot above the heatmap represents the number of mutated genes identified per procedure, while the bar plot on the right shows the overall frequency of mutations for each gene across the cohort. Annotated bars above the heatmap indicate, from top to bottom: definitive diagnosis (red for at least high-grade dysplasia, green for benign); diagnostic method used to establish the definitive diagnosis (cytohistology, clinical evolution, endoscopy, imaging shown in a red gradient, and clinical evolution with or without additional methods shown in light grey); presence of primary sclerosing cholangitis (blue); DNA qualification (yes in grey, no in black); type of endoscopic lesion (stricture, stricture with mass, mass, or inflammatory lesion shown in a pink gradient); and cytohistological category (no morphological abnormality, reactive changes, indeterminate for dysplasia, low-grade dysplasia shown in a green gradient, and high-grade dysplasia/*in situ* carcinoma, invasive carcinoma shown in an orange gradient). ERCP, endoscopic retrograde cholangiopancreatography; VUS, variant of unknown significance.

Definitive diagnoses identified malignant causes in 76 patients (73.1%) and non-malignant causes in 28 patients (26.9%). Definitive diagnosis of malignancy was established based on cytohistological examination (from ERCP, metastasis biopsy or surgical resection), clinical evolution, imaging, and/or endoscopic findings (Fig. 2, Table S2). In non-malignant cases, definitive diagnosis relied primarily on clinical evolution, either alone or in combination with additional diagnostic methods, including cytohistological examination, imaging, endoscopy, biological analyses, constitutional genetics, and exploratory surgery (Fig. 2, Table S2). The mean time from the first sampling procedure to definitive diagnosis for the overall cohort was 2.7 months (range 0–45.3). This interval was shorter in patients with malignant cause (mean 1.9 months, range 0–45.3) compared with those with non-malignant cause (mean 5.1 months, range 0–24). Fig. S1 depicts the initial lesion/stricture classification (suspect, indeterminate, not suspect), the time from the first sampling procedure to definitive diagnosis, as well as the duration of follow-up, for all patients.

Among patients with a definitive diagnosis of malignancy, clinical management was guided by multidisciplinary discussion and included surgical resection, with or without adjuvant chemotherapy, radiochemotherapy, systemic therapy (chemotherapy with or without immunotherapy), or best supportive care, as appropriate (Table S2). All patients who underwent surgical resection (n = 20) had malignancy confirmed on the resection specimen.

In total, 445 samples were collected, with 265 biliary brushings and 180 biliary biopsies, including 165 biopsies obtained using the SpyBite™ forceps (Fig. S2).

### Cytohistological examination

**Fig. 3:**
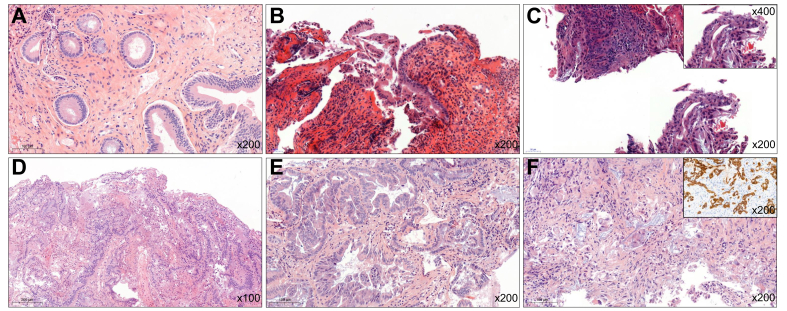
Representative examples of each cytohistological diagnostic category (A) No morphological abnormalities (H&E stain, x200); (B) Reactive changes with acute inflammation (H&E, x200); (C) Lesion indeterminate for dysplasia in an inflammatory context (H&E, x200 and inset: x400); (D) Intraductal papillary neoplasm with low-grade dysplasia (H&E, x200); (E) Biliary high-grade dysplasia (H&E, x200); (F) Invasive cholangiocarcinoma (H&E, x200 and inset: cytokeratin 7 immunohistochemistry, x200).

Samples collected for cytohistological evaluation from the 127 procedures included biliary brushings alone in 22 procedures (17.3%), biliary biopsies alone in six procedures (4.7%), and both biliary brushings and biopsies in 99 procedures (78%). Cytohistological analysis revealed: nine procedures (7.1%) without morphological abnormalities, 37 procedures (29.1%) with reactive changes, 18 procedures (14.2%) with lesions indeterminate for dysplasia, 11 procedures (8.6%) with low-grade dysplasia, 17 procedures (13.4%) with high-grade dysplasia or *in situ* carcinoma, and 35 procedures (27.6%) with invasive carcinoma. Representative images for each category are available in Fig. 3.

For the 90 procedures with a definitive diagnosis of malignancy, 52 (57.8%) were positive for malignancy and 38 (42.2%) were negative for malignancy according to the cytohistological examination. All 37 procedures with a definitive diagnosis of non-malignancy were negative for malignancy according to the cytohistological examination (Fig. 2).

### DNA qualification and sequencing

To determine the most suitable procedure for DNA sequencing, we evaluated the DNA quality of two types of samples, biliary brushings and biopsies, preserved in PreservCyt® and in RNAprotect® Cell Reagent. Higher DNA quality was obtained from brushings compared to biopsy supernatants (*p* = 1.4x10^-32^), while no difference was observed between brushing samples preserved in PreservCyt® and RNAprotect® Cell Reagent (*p* = 1) (Fig. S3). Thus, DNA from biopsy supernatants was discontinued for use after the 54th procedure (except for one procedure where only a biopsy sample was available).

The flow chart in Fig. S2 shows the number of samples, procedures, and patients throughout the workflow, including collection during ERCP, DNA quality assessment, sequencing success, and identification of pathogenic variants. In total, 279/445 samples (62.7%) from 121 procedures (95.3%) were subjected to DNA sequencing. Six procedures (4.7%), including four limited only to biopsy specimens, did not yield qualified DNA. Pathogenic variants were identified in 71 procedures (55.9%), a singular VUS was identified in one procedure (0.8%) and no mutation was detected in 49 procedures (38.6%).

The correlation between endoscopic findings, cytohistological classification, DNA qualification for sequencing, and genomic alterations, along with the definitive diagnosis for each procedure is represented in Fig. 2. For the 86 procedures with a definitive diagnosis of malignancy and available material for DNA sequencing, 70 (81.4%) were classified as pathogenic, one (1.2%) was classified as VUS and 15 (17.4%) were classified as wild type. The same targeted NGS was additionally performed on surgical specimens in 7/20 patients who underwent surgical resection with confirmation of cancer and showed concordance with the molecular alterations identified in ERCP-derived samples in six cases. In one patient (P064), no variants were detected in biliary brushing samples, whereas NGS performed on the surgical resection revealed pathogenic mutations in *KRAS*, *TP53*, *SMAD4* and *CDKN2A*. For the 35 procedures with a definitive diagnosis of non-malignancy and available material for DNA sequencing, pathogenic *KRAS* variants were identified for one (2.9%), and 34 (97.1%) were classified as wild type (Fig. 2).

### FISH analysis

A FISH analysis was performed retrospectively on a subgroup of 47 procedures from 42 patients. These procedures were selected based on cytohistological results and availability of material: one procedure without morphological abnormalities, eight with reactive changes, 11 with lesions indeterminate for dysplasia, two with low-grade dysplasia, six with high-grade dysplasia/*in situ* carcinoma, and 18 with invasive carcinoma. FISH was applied on 41 biopsy specimens and six cell blocks. Among these, 41 procedures (87.2%) had a definitive diagnosis of malignancy, while six procedures (12.8%) were diagnosed with non-malignant conditions.

FISH was positive for 31 procedures: 23 with polysomy (47.9%), including five with concurrent 9p21 loss (Fig. S4B and C), seven with trisomy 3 (16.7%), including two with concurrent 9p21 loss, and one with trisomy 7 (2.1%). Eight procedures showed no significant anomalies (16.7%) (Fig. S4A), and eight were uninterpretable (16.7%): four due to excessive nuclear overlap, three due to insufficient cellular material (<25 cells), and one due to hybridization failure. Detailed results for each procedure are provided in Table S2.

### Comparison of the diagnostic performances of cytohistology, NGS and FISH

All results of the diagnostic performances of each diagnostic modalities for the overall cohort and the subgroup with FISH analysis are summarized in Table 2.

**Table 2:** Diagnostic performances of different diagnostic modalities.

Diagnostic modality	Se [95% CI]	Sp [95% CI]	PPV [95% CI]	NPV [95% CI]	Accuracy
**Overall cohort (N = 104 patients)**					
Cytohistology	59.2% [0.48–0.70]	100% [0.89–1.00]	100% [0.93–1.00]	47.5% [0.35–0.60]	70.2%
NGS	82.2% [0.73–0.91]	96.2% [0.89–1.00]	98.4% [0.95–1.00]	65.8% [0.51–0.81]	85.9%
Cytohistology + NGS	89.5% [0.83–0.96]	96.4% [0.90–1.00]	98.6% [0.96–1.00]	77.1% [0.63–0.91]	91.3%
**Subgroup (n** = **42 patients)**					
Cytohistology	66.7% [0.51–0.82]	100% [0.50–1.00]	100% [0.88–1.00]	33.3% [0.12–0.55]	71.4%
NGS	86.1% [0.75–0.97]	100% [0.50–1.00]	100% [0.90–1.00]	54.5% [0.25–0.84]	88.1%
Cytohistology + NGS	97.2% [0.92–1.00]	100% [0.50–1.00]	100% [0.91–1.00]	85.7% [0.60–1.00]	97.6%
FISH	81.2% [0.68–0.95]	50% [0.01–0.99]	92.9% [0.83–1.00]	25% [0.00–0.55]	77.8%
Cytohistology + FISH	86.1% [0.75–0.97]	66.7% [0.29–1.00]	93.9% [0.86–1.00]	44.4% [0.12–0.77]	83.3%

FISH, fluorescence *in situ* hybridization; NGS, next-generation sequencing; NPV, negative predictive value; PPV, positive predictive value; Se, sensitivity; Sp, specificity. Se, Sp, PPV, and NPV were calculated retrospectively for each test relative to the definitive diagnosis using standard 2×2 contingency tables.

In the overall cohort (n = 104 patients), the diagnostic performances of NGS were significantly better than cytohistological examination with a better sensitivity for detecting at least high-grade dysplasia, a high specificity (*p* = 1.9x10^-3^), a high PPV (*p* = 0.3) and a better NPV (*p* = 9.5x10^-5^). The combination of NGS and cytohistological assessment demonstrated even better performances with a better sensitivity, similar Sp (*p* = 4.1x10^-2^) and PPV (*p* = 0.4) and a better NPV (*p* = 1.4x10^-2^).

In the subgroup with FISH (n = 42 patients), FISH showed slightly lower sensitivity than NGS but higher than cytohistology. However, its specificity and NPV were notably lower than those of both NGS and cytohistology. In terms of overall diagnostic accuracy, NGS, and particularly the combination of NGS and cytohistology, outperformed FISH alone and the combination of cytohistology and FISH.

### Subanalysis of patients with primary sclerosing cholangitis

In total, 13 patients had PSC: 11 with a prior diagnosis and 2 newly diagnosed during the study. Six of them underwent repeated procedures (2–8 per patient). They all exhibited EBS. After a mean follow-up period of 22.7 months (95% CI 12-33.4), one patient developed CCA, and one patient developed high-grade biliary dysplasia (*P102* and *P004*). The remaining 11 patients exhibited only reactive cytohistological changes. No molecular alterations were detected in this group, except in one patient (P089) in whom a pathogenic *KRAS* variant was identified in one sample (p.(Gly12Asp), with a low variant allele frequency of 2.12% (Fig. 2). All 11 patients were ultimately classified as having non-complicated PSC as the definitive diagnosis. Patient P089, for whom no histological evidence of malignancy was found, was referred for liver transplantation because of recurrent episodes of cholangitis.

**Fig. 4:**
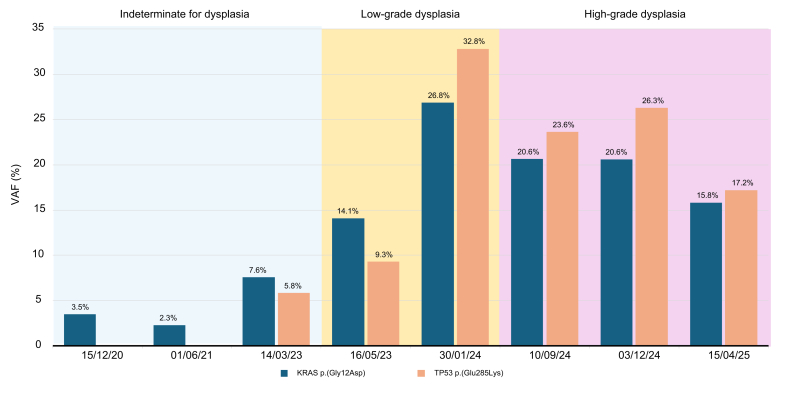
Evolution of VAF over time in a patient (P004) with PSC. The plot depicts the VAF of two pathogenic variants, *KRAS* p.(Gly12Asp) (shown in blue) and *TP53* p.(Glu285Lys) (shown in orange), measured in one representative sample from each of eight ERCP procedures performed between 2020 and 2025. Each time point corresponds to a distinct procedure. Background shading indicates the cytohistological assessment at each procedure (blue: indeterminate for dysplasia; yellow: low-grade dysplasia; pink: high-grade dysplasia), illustrating the temporal relationship between molecular alterations and pathological evaluation. ERCP, endoscopic retrograde cholangiopancreatography; PSC, primary sclerosing cholangitis; VAF, variant allele frequency.

One patient who developed high-grade biliary dysplasia on PSC underwent eight endoscopic procedures between December 2020 and April 2025 (*P004*). Cytohistological evaluation revealed a progression of biliary dysplasia over time on the common bile duct as shown in Fig. 4. Notably, DNA sequencing identified oncogenic alterations before cytohistological confirmation of neoplasia. A pathogenic *KRAS* mutation was detected early in the course, followed by the emergence of a pathogenic *TP53* mutation 2 years later, with increasing variant allele frequencies over successive procedures. Thus, molecular alterations preceded the cytohistological confirmation of neoplasia by more than 4 years. Following multidisciplinary team discussion, the patient was referred for liver transplantation.

### DNA molecular profiling of malignancies

A total of 76 patients had a definitive diagnosis of malignancy, including 55 CCA, eight pancreatic adenocarcinomas (ADK), three ampullary adenoma with high-grade dysplasia/ADK, three gallbladder ADK, two intraductal papillary neoplasm of the bile duct with high-grade dysplasia, one biliary high-grade dysplasia (on PSC), one duodenal ADK, one hepatocellular carcinoma, one neuroendocrine carcinoma of the main bile duct, and one extrinsic compression by carcinomatosis (Table 1). DNA molecular profiling was successfully performed for 73 patients (96.1%).

Among the biliary cancers (CCA, gallbladder ADK, intraductal papillary neoplasm of the bile duct with high-grade dysplasia and biliary high-grade dysplasia developed on PSC), three CCA (4.9%) did not yield qualified DNA and 47 (77%) harbored at least one pathogenic variant (Fig. 2). The most common alterations included mutations in *TP53* (n = 27), *KRAS* (n = 23), *SMAD4* (n = 12), and *CDKN2A* (n = 11). Less frequent pathogenic alterations included mutations in *BRAF* (n = 4), including one targetable *BRAF*^V600E^ mutation, *ERBB2* (n = 4), *BRCA2* (n = 2), *STK11* (n = 2), *PIK3CA* (n = 2), *BRCA1* (n = 1), *CTNNB1* (n = 1), and *NRAS* (n = 1).

Copy number analysis was retrospectively performed in 27 patients with a definitive diagnosis of malignancy, including 21 CCA, two pancreatic ADK, one gallbladder ADK, one intraductal papillary neoplasm of the bile duct with high-grade dysplasia, one biliary high-grade dysplasia (on PSC), and one case of carcinomatosis. Copy number alterations were identified in two patients. One patient with CCA (P059) exhibited *ERBB2* amplification (7–8 copies) in both analyzed samples (one brushing preserved in PreservCyt® and one in RNAprotect® Cell Reagent). Immunohistochemistry showed HER2 2+ expression, and *in situ* hybridization confirmed true amplification. Another patient with gallbladder ADK exhibited *KRAS* amplification (7 copies) in both analyzed samples, of unknown clinical significance.

To further assess HER2 status, immunohistochemistry with or without *in situ* hybridization was performed when sufficient material was available. Among 55 patients with CCA and three with gallbladder ADK, HER2 status was assessable in 38 patients. Except for patient P059, none demonstrated HER2 positivity (23 scored 0, four scored 1+, and 10 scored 2+ without amplification by *in situ* hybridization).

### Exploratory RNA sequencing analysis

To evaluate the feasibility of RNA molecular profiling, a subset of 16 procedures was selected retrospectively based on a definitive diagnosis of malignancy and availability of material (14 CCA, 1 gallbladder ADK, and 1 pancreatic ADK). RNA fusion analysis was performed on 28 samples derived from these 16 procedures. One *EGFR* (ENSG00000146648) rearrangement of unknown significance (*7:55151362::7:54603740*) was identified in a CCA sample and subsequently confirmed in the corresponding surgical specimen. No fusion transcripts were detected in 12 samples, while 15 samples (53.6%) were non-contributory due to insufficient RNA quality or quantity based on our quality control metrics.

## Discussion

EBS pose a significant diagnostic challenge due to the difficulty in obtaining adequate tissue samples for pathological assessment, the broad spectrum of differential diagnoses, including both malignant and benign etiologies, and the urgent need for timely management in cases of CCA. Despite advances in endoscopic and cytohistological techniques, diagnostic performances remain insufficient^7^^,^^11^^,^^20^ and ERCP with cholangioscopy-directed biopsies has only moderately improved these diagnostic performances, with sensitivity rates between 60.1% and 90% depending on the generation of the system. Notably, in PSC cases, SOC has achieved sensitivities of 33–65%.[6], [7], [8], [9], [10]

In this prospective study, we evaluated the contribution of targeted NGS to the diagnosis of EBS using samples obtained via ERCP and SOC with the SpyGlass™ system in patients with indeterminate EBS or suspected eCCA. To the best of our knowledge, this is the first study to focus exclusively on extrahepatic samples obtained through an optimized procedure using the SpyGlass™ system and evaluating the integration of NGS to this procedure. Our findings demonstrate that combining cytohistological examination with targeted DNA sequencing improves diagnostic accuracy, reaching up to 91.3%. This outperforms cytohistology alone (70.2%) and targeted NGS alone (85.9%). Moreover, our results surpass those reported in previous studies, where the sensitivity of NGS alone ranged from 73% to 75%, compared to 82.2% in our study, and the combined sensitivity of NGS and cytohistology reached 89.5%, exceeding the previously reported range of 83% to 85.7%.^14^^,^^16^^,^^21^ Additionally, our method gives better results obtained with less material waste, thus limiting invasive procedures (multiple sampling) and preserving cytological and histological material for other analyses for diagnostic or theranostic purposes. With our protocol, we showed that only a few milliliters of the preservative solution used for brushings are sufficient for both DNA and RNA sequencing. As brushings are typically performed during the initial procedure, our results indicate that additional, more invasive procedures, particularly dedicated biopsies for molecular analysis, are unnecessary. Furthermore, using brushings rather than biopsies may reduce the risk of false negatives due to intratumoral heterogeneity, as the sample is more likely to contain genetic material shed by tumor cells along the biliary tract. With our optimized procedure, only six procedures (4.7%) were not contributive for DNA sequencing. Furthermore, we observed equal performances for both preservative solutions, PreservCyt® and RNAprotect® Cell Reagent.

In addition, we explored the feasibility of targeted RNA sequencing from malignant samples obtained via ERCP and SOC using the SpyGlass™ system. Although RNA sequencing was technically successful in a subset of samples and enabled the identification of a fusion transcript in one case, the overall contribution of RNA molecular profiling in this study was limited. More than half of the samples were non-contributory (53.6%) due to insufficient RNA quality or quantity, regardless of the preservative solution used. These findings highlight the current technical challenges associated with RNA-based analyses in this context and indicate that further optimization of the method is still required.

We next compared the diagnostic performance of multicolor FISH using the UroVysion® assay to cytohistological examination and NGS. FISH has emerged as a promising tool to aid in the diagnosis of indeterminate biliary strictures, with sensitivity up to 61% but with disparate results.^11^^,^^18^^,^^22^ However, it remains infrequently used in routine clinical practice and is not currently recommended.^1^ FISH analysis was performed on a subgroup of 42 patients. Although it showed good sensitivity (81.2%), its specificity (50%) and NPV (25%) were low. Overall accuracy was moderate (77.8%), lower than that of NGS alone (88.1%) and markedly lower than the combination of cytohistology and NGS (97.6%). In addition, 16.7% of cases were uninterpretable. Multicolor FISH remains technically challenging and labor-intensive. As previously reported, we encountered interpretation difficulties due to frequent nuclear overlap, particularly in inflammatory contexts, which complicated probe signal enumeration. Although FISH is often considered a more affordable alternative to NGS, the growing accessibility of NGS in routine clinical practice and the progressive reduction of its cost is challenging this assumption. While NGS remains costly, its use at the time of diagnosis enhances diagnostic accuracy, potentially reducing the need for repeat procedures. Additionally, earlier diagnosis could facilitate timely therapeutic interventions and help avoid unnecessary healthcare costs associated with diagnostic delays, ultimately offering a favorable cost-benefit balance.

A major diagnostic challenge is PSC, and particularly biliary neoplasia developed on PSC, as the risk of malignancy is high and cytohistological diagnosis difficult due to important inflammation. Moreover, radical surgery or liver transplantation are the only curative treatment options but must be performed at an early stage. Three major studies focusing on patients with PSC showed the clinical utility of NGS in this context, with improved sensitivities of up to 75%, which were further improved by the combination with morphological analysis.[23], [24], [25] In our study, 13 patients (12.5%) had PSC, and molecular alterations were detected in both patients with a definitive diagnosis of high-grade dysplasia/CCA. Our findings suggest that molecular profiling may allow for the detection of early oncogenic alterations in PSC-associated biliary strictures, even when cytohistological analysis remains non-diagnostic, with potential early curative treatment. However, these results must be interpreted with caution, as the PSC subgroup was small (n = 13), limiting the strength of our conclusions. Confirmation in larger, prospective PSC cohorts will be required.

Implementing NGS at the time of diagnosis not only improves diagnostic accuracy but also enables rapid molecular profiling, allowing for the identification of therapeutic targets in a single procedure. NGS is a highly sensitive technology capable of performing multigene analysis, making it particularly suitable for these low-cellularity samples, especially given that theses samples are often the only material available as fewer than 35% of eCCA are eligible for surgical resection.^26^ In addition, molecular profiling of CCA using FFPE biopsies is often challenging, with reported sequencing failure rates of up to 25%, and even higher rates observed for gene fusion detection.^27^ While data on NGS of circulating tumor DNA in eCCA is limited, available studies report detection rates of genomic alterations in approximately 55% of cases.^28^

In our study, DNA sequencing was successfully performed for 96.1% of malignancies (CCA or other carcinomas), far exceeding the sequencing success rates from other types of material. Although our panel was not specifically developed for CCA (pan-tumor panel of 50 genes), it includes genes of interest for CCA treatment such as *IDH1*, *BRAF* and *ERBB2*. In addition, we explored the feasibility of molecular profiling using RNA extracted from these samples with a custom-designed targeted panel and demonstrated its applicability, supporting its potential as a screening tool for personalized therapeutic approaches.^29^

One major limitation of NGS is the risk of false positives and over-diagnosis, potentially leading to inappropriate treatment. Oncogenesis, particularly in the context of PSC, remains poorly understood.[30], [31], [32] The sequence of molecular alterations involved in malignant transformation, and their correlation with morphological features, are not yet clearly defined. *KRAS* mutations appear to be an early event in neoplastic progression, but are insufficient on their own to drive malignant transformation.^33^^,^^34^ Moreover, *KRAS* mutations can also be detected in non-malignant conditions such as autoimmune pancreatitis.^35^ In contrast, *TP53* alterations seem to be a late event, associated with malignant transformation.^34^ Therefore, the identification of a single molecular alteration, particularly a *KRAS* mutation, is not sufficient to establish a diagnosis of malignancy. Correlation with morphological and clinical data remains essential. Similarly, the detection of molecular alterations at low allele frequencies, such as *KRAS* mutations, must be interpreted with caution, particularly when using highly sensitive NGS techniques. This concern is especially relevant in the context of cell-free DNA sequencing. Recent studies demonstrated the diagnostic potential of cell-free DNA in bile, given the ease of bile collection during ERCP, including in the context of PSC. While this approach showed excellent sensitivity (96.4%), its specificity was lower than that observed in our study (69.2%). This was attributable to the detection of mutations in patients ultimately diagnosed with benign disease.^36^^,^^37^ This may reflect true false-positive results or the detection of precancerous or early-stage malignant lesions, which will require further investigation. Other biomarkers, such as aberrant DNA methylation, seem promising,[38], [39], [40] but these technologies are not yet recommended for routine clinical practice.^1^

Our study has several limitations. First, the targeted panel used was not specifically designed for eCCA and lacked several relevant genes of interest (*ELF3*, *ARID1B*, *PBRM1*, *BAP1*), which may have led to false-negative results and affected diagnostic sensitivity. On the other hand, a smaller panel is more accessible to most laboratories and therefore more readily applicable. Moreover, although we were able to retrospectively incorporate copy number variation analysis in a subset of patients, this approach was not applicable to the entire cohort and identified only one clinically actionable *ERBB2* amplification. Consequently, the absence of systematic copy number analysis in all patients remains an important limitation. Second, the number of patients followed for strictures in the context of PSC was insufficient to accurately characterize the sequence of molecular alterations and assess their true diagnostic value. Third, the complexity and potential risk of the procedure raise practical questions, particularly regarding whether an additional brushing dedicated solely to molecular analyses should be routinely performed. Fourth, while our approach demonstrated strong diagnostic performance, it was conducted using the SpyGlass™ system, which is not commonly employed as a first-line diagnostic tool. However, the EASL guidelines acknowledge its use in the initial ERCP can be cost effective in expert centers.^1^ Fifth, although technically feasible, RNA sequencing showed a high failure rate and limited added diagnostic or therapeutic value in this study. Given the low proportion of contributory samples and the small number of patients analyzed, RNA sequencing from ERCP-derived material cannot currently be recommended for routine clinical use (avoiding waste of material) and will require further methodological optimization. This is notably an important limitation as *FGFR2* fusions, a rare but important therapeutic target in eCCA, could not be analyzed in our study. Finally, the actual impact on therapeutic decision-making, potential time-saving in diagnosis, and cost-effectiveness of this strategy remain to be determined.

In conclusion, our findings support the clinical value of integrating NGS with cytohistological evaluation of extrahepatic biliary specimens obtained through SOC. This combined approach not only improves diagnostic performances but also facilitates the early identification of targetable genomic alterations at the time of diagnosis, therefore optimizing therapeutic decision-making. An implication of these results is the need to reconsider and potentially redefine surveillance strategies for EBS, particularly for patients lacking morphological evidence of malignancy but harboring pathogenic variants.

## Abbreviations

ADK, adenocarcinoma; CCA, cholangiocarcinoma; cfDNA, cell-free DNA; EBS, extrahepatic biliary strictures; eCCA, extrahepatic cholangiocarcinoma; ERCP, endoscopic retrograde cholangiopancreatography; FISH, fluorescence *in situ* hybridization; iCCA, intrahepatic cholangiocarcinoma; NGS, next-generation sequencing; NPV, negative predictive value; PPV, positive predictive value; PSC, primary sclerosing cholangitis; SOC, single-operator cholangioscopy; VUS, variant of unknown significance.

## Authors’ contributions

JS, JMP, CB and ACB designed the study. AC, KB, LB and JMP performed endoscopic procedures. JS and ACB performed cytohistological examination. ACB and JS performed FISH analyses. ACB, JS, HR, DG, RG and SE performed molecular analyses. AC, KB, LB, JMP, NF and EA followed the patients. ACB collected the data. ACB and HR performed statistical analyses. ACB, JMP and JS wrote the article. All authors reviewed the article.

## Data availability

The authors declare that the data presented in this article are available as raw data (raw data files and protocols) upon request to the corresponding author.

## Declaration of generative AI and AI-assisted technologies in the writing process

During the preparation of this work the authors used ChatGPT in order to improve language and readability only. After using this service, the authors reviewed and edited the content as needed and take full responsibility for the content of the publication.

## Financial support

No financial support was received to produce this manuscript.

## Conflict of interest

Nothing to report for all authors.

Please refer to the accompanying ICMJE disclosure forms for further details.
