# Supplementary material for: Molecular endoscopy with next-generation sequencing improves diagnosis of cholangiocarcinoma in patients with extrahepatic biliary strictures
Source: JHEP Rep. 2026 Feb 16;8(5):101788. doi: 10.1016/j.jhepr.2026.101788 (PMC13058966; doi:10.1016/j.jhepr.2026.101788)
Supplement: Multimedia component 1 [file mmc1.pdf]

Molecular endoscopy with next-generation sequencing improves diagnosis of cholangiocarcinoma in patients with extrahepatic biliary strictures

Anne-Cécile Brunac, Adrian Culetto, Hadrien Reboul, Karl Barange, Louis Buscail, Nadim Fares, Ronan Guillemin, Emily Alouani, David Grand, Solène Evrard, Céline Basset, Jean-Marie Peron, Janick Selves

Table of contents

Supplementary methods……………………………………………………………….2

Fig. S1…………………………………………………………………..……...……..5

Fig. S2…………………………………………………………………...……..….6

Fig. S3…………………………………………………………………….....……7

Fig. S4…………………………………………………………………….……...…..8

Table S1………………………………………………………………….…………...9

Table S2 legend……………………………………………………………………10

Supplementary references………………………………………………….…….…11

Supplementary methods

Sampling and sample processing

Procedures were performed by experienced endoscopists (duodenoscope: Evis Exera III TJF-Q190V, Olympus Medical Systems Corp, Japan; cholangioscope: SpyScope™ DS II M00546610, Boston Scientific, USA). Biliary brushings (Biliary Cytology Brush FS-CB-1.5-5, Cook Medical, USA) and biopsies, including biopsies obtained with SpyBite™ forceps (SpyBite™ Max 1.2mm 286cm M00546470, Boston Scientific, Costa Rica) were collected. Concerning the two types of preservative solutions, PreservCyt® solution (Hologic Corp., USA), is commonly used for cytological analysis. RNAprotect® Cell Reagent (Qiagen, Germany) is a medium validated within the BACAP protocol (Biological and Clinical Database for Pancreatic Adenocarcinoma) (NCT02818829) (1). Two brush passes were performed per procedure. The first brush was immersed in 20 mL of PreservCyt®, and the second brush in 25 mL of RNAprotect® Cell Reagent. Brushes were agitated in their respective vials to detach cells, after which they were removed, and the vials transported to the pathology laboratory. Biopsies collected with SpyBite™ forceps were immersed in 20 mL of PreservCyt® solution.

In the laboratory, brushing samples immersed in PreservCyt® solution were divided, with 16 mL allocated for cytological analysis (cell blocks) and 4 mL reserved for molecular analyses. Samples immersed in RNAProtect® Cell Reagent were used exclusively for molecular analyses. Cell pellets were prepared within 24 hours of sample collection, aliquoted, and stored at -80°C until DNA and RNA extraction. Biopsy specimens were fixed in formaldehyde and embedded in paraffin for pathological examination. Cell pellets obtained from the supernatants were processed for molecular analyses.

DNA and RNA sequencing

Simultaneous extraction of genomic DNA and RNA was performed using the AllPrep DNA/RNA/miRNA Universal Kit (Qiagen, 80224), in accordance with the manufacturer's protocol. Extracted DNA and RNA were quantified with Qubit Fluorometer (Thermo Fisher Scientific). DNA quality was evaluated by qPCR using an in-house protocol (2). Qualified DNA was sheared using a Covaris® ME220 system.

Library preparation was performed with the Twist Library Preparation Kit, Mechanical Fragmentation (104177) using the KAPA HiFi HotStart ReadyMix DNA polymerase (Roche sequencing, KK2602), followed by capture enrichment using NGS Target Enrichment Solutions (Twist Bioscience). Sequencing was performed on an Illumina® NextSeq 550Dx in paired-end sequencing (2x149 cycles) (San Diego, CA). Our custom-designed panel targets 50 genes (Supplementary Table 1). We target a minimum depth of 150X on each targeted nucleotide and this threshold must be achieved on at least 97% of targeted nucleotides to validate the results. Data were analyzed using an in-house pipeline. Briefly, reads were aligned on a human genome (GRCh38) with BWA mem (v0.7.17) software, and variant calling was conducted with FreeBayes (v1.2.0), Mutect2 (v4.1.4.1) and VarDict-java (1.7.0). The assay's detection limit was 2% mutant allele frequency. Variants were classified based on the classification for somatic alterations in solid tumors (3). CNV were called using CNVkit (v0.9.11) and an in-house software based on Z-scores of normalized depth and allele frequency.

RNA samples underwent rRNA depletion using the KAPA RNA HyperPrep Kit with RiboErase (HMR) (Roche sequencing, 07962274001) to generate whole transcriptome stranded libraries, indexed with the unique dual Index adapter system (Twist, Bioscience, 101308). Whole transcriptome libraries were subjected to a capture enrichment step and sequenced as described for DNA. Our custom-designed panel targets 98 genes, including *FGFR2* (Supplementary Table 1). Capture allows the genes of interest from the panel to be recovered, as well as, by overflow, the translocation zone of their partners, even if they are not themselves in the panel. Fusion transcripts were detected using STAR fusion integrated into an in-house pipeline (4,5).

HER2 status evaluation :

HER2 immunohistochemistry was performed with 4B5 monoclonal antibody (05999570001, ROCHE) and *in situ* hybridization using VENTANA HER2 Dual ISH DNA Probe (08314373001, ROCHE), both on Benchmark Ultra platform. VENTANA HER2 Dual ISH DNA Probe Cocktail contains HER2 probes and Chromosome 17 probes. Results are reported as a ratio of HER2/Chromosome 17 to determine HER2

amplification status (HER2/Chromosome 17 ratio ≥ 2.0 is amplified, while a ratio < 2.0 is non-amplified).

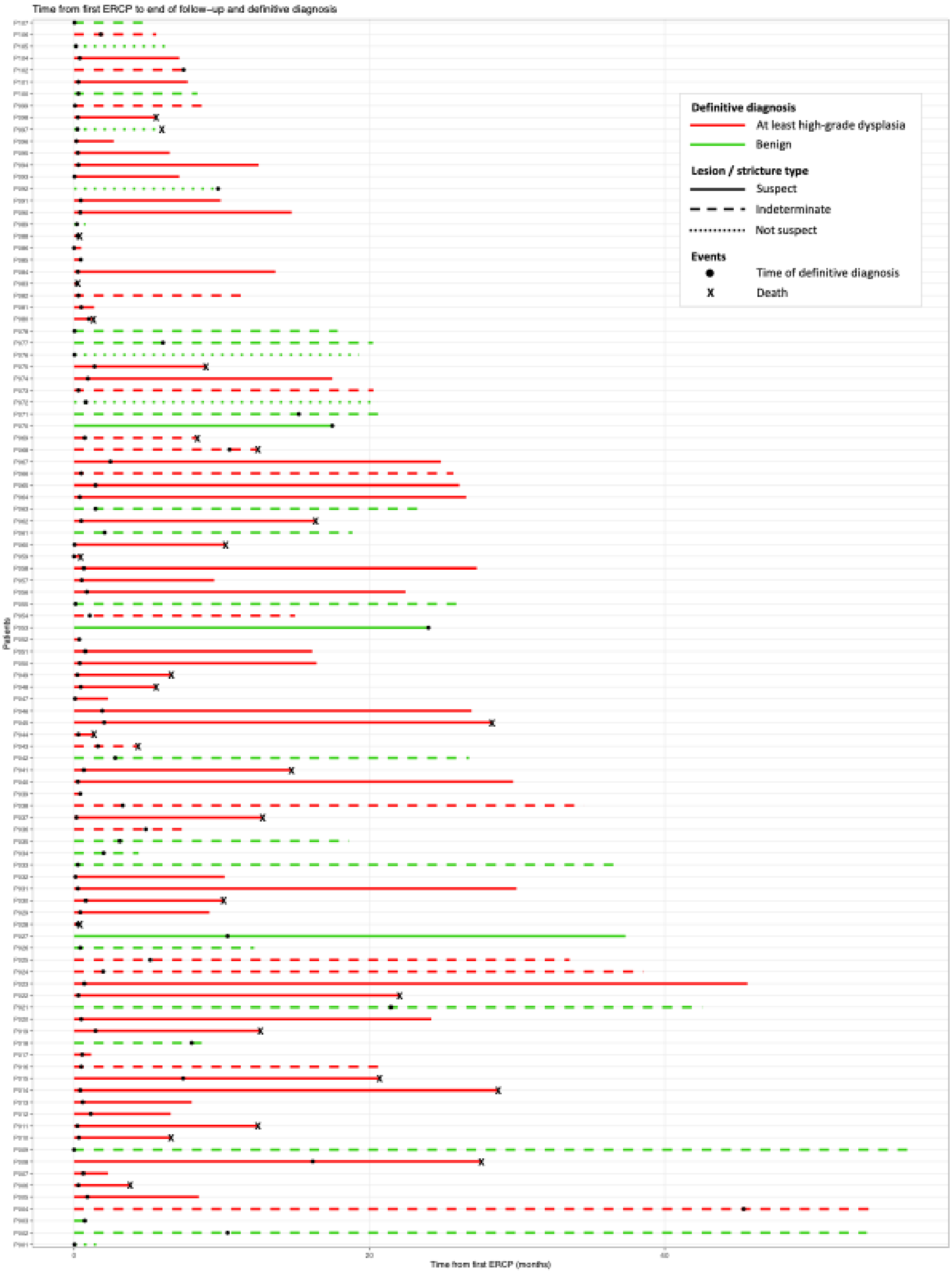

Fig. S1. Swimmer plot illustrating the time from the first sampling procedure to definitive diagnosis, along with lesion/stricture characteristics, duration of follow-up, and occurrence of death, where applicable, for all 104 patients.

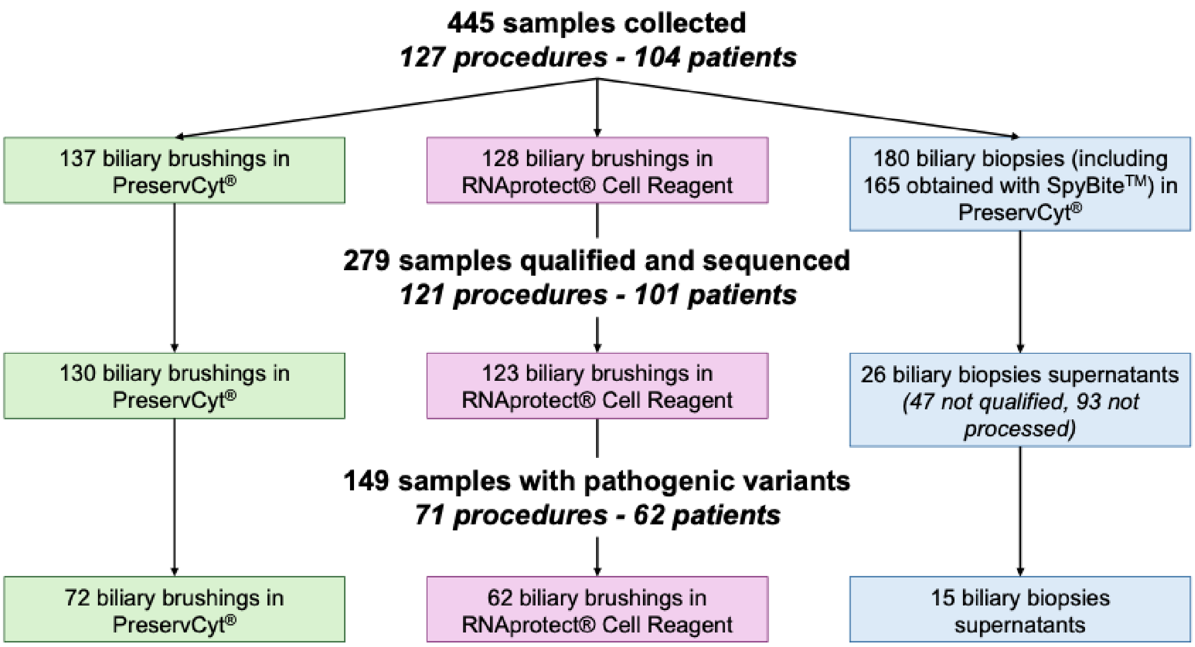

Fig. S2. Flow chart with the number of samples, procedures, and patients throughout the workflow, including collection during ERCP, DNA quality assessment, sequencing success, and identification of pathogenic variants.

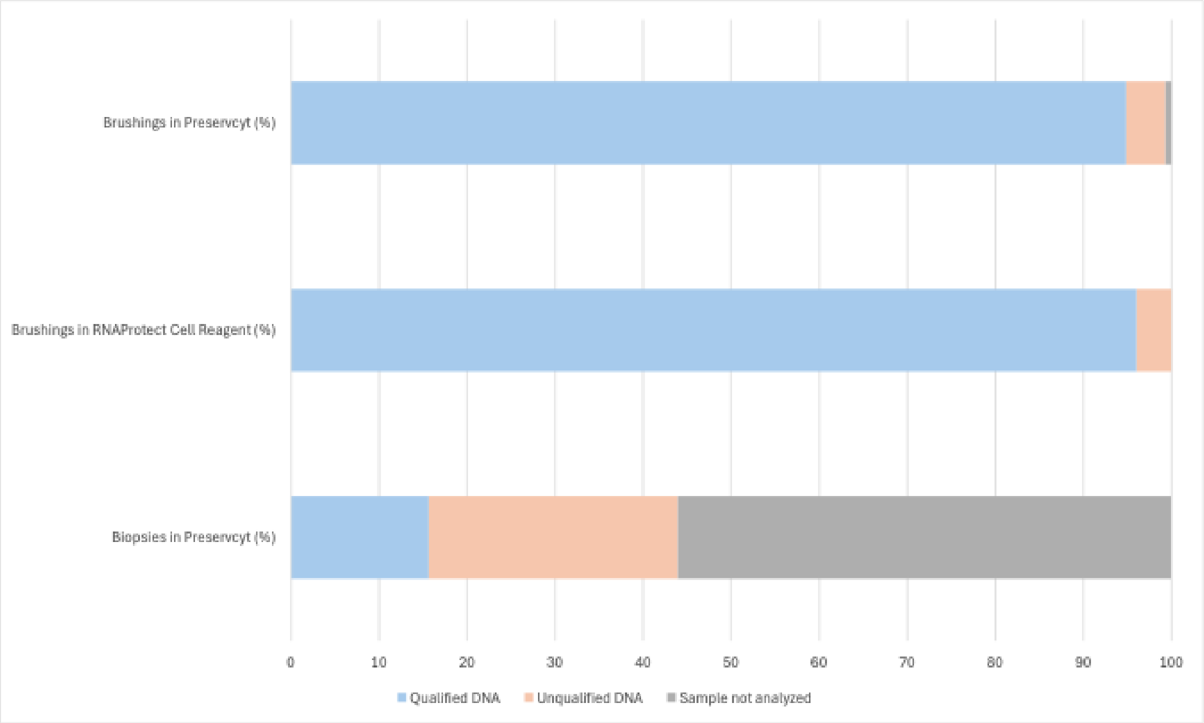

Fig. S3. DNA quality assessed by qPCR for the 3 types of samples. The quality of the DNA was assessed by qPCR and categorized as qualified (blue) or non-qualified (orange). 1 DNA sample from brushings in Preservcyt® (0.7%) and 93 DNA samples from biopsy supernatants (56%) were not analyzed (grey).

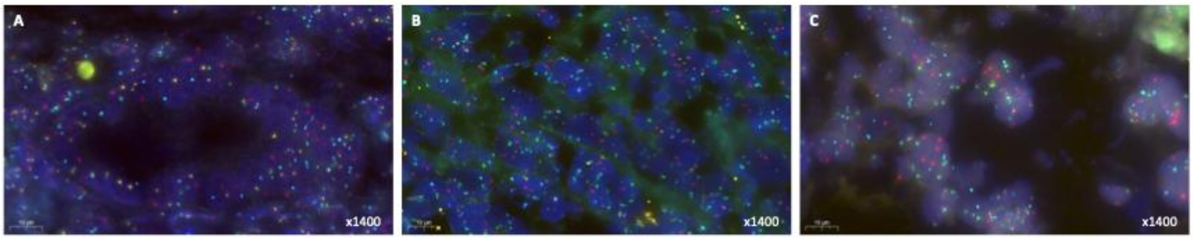

Fig. S4. Fluorescence in situ hybridization using the UroVysion® assay. (A) Procedure with normal disomic cells (two signals) for chromosomes 3 (red), 7 (green), 17 (blue), and 9p21 (yellow); (B) Procedure with polysomy; (C) Procedure with polysomy and concurrent homozygous 9p21 loss.

Table S1. Gene lists from the solid tumors panel for targeted DNA sequencing (left) and for targeted RNA sequencing (right). The 13 genes shown in bold are those for which copy number variation (CNV) analysis is feasible, as all exons or coding sequences are covered by the panel.

**DNA panel**		**RNA panel**			
ACVR1	*HRAS*	*ACTB*	*FGFR1*	*NCOA2*	*PRKD1*
AKT1	*IDH1*	*ALK*	*FGFR2*	*NF1*	*PRKD2*
ALK	*IDH2*	*ARHGAP26*	*FGFR3*	*NFIB*	*PRKD3*
BRAF	*JAK2*	*ASPSCR1*	*FN1*	*NOTCH1*	*RAF1*
BRCA1	*KIT*	*ATF1*	*FOS*	*NOTCH2*	*RELA*
BRCA2	*KRAS*	*BCOR*	*FOSB*	*NOTCH3*	*RET*
CDKN2A	*MAP2K1*	*BRAF*	*FOXO1*	*NOTCH4*	*ROS1*
CTNNB1	***MET***	*C11orf95*	*FOXR2*	*NR4A3*	*SMARCB1*
DICER1	***MYC***	*CAMTA1*	*FUS*	*NRG1*	*SRF*
DDR2	***MYCN***	*CDKN2A*	*GNAS*	*NTRK1*	*SS18*
EGFR	*MYOD1*	*CIC*	*GRM1*	*NTRK2*	*STAT6*
ERBB2	*NRAS*	*CITED1*	*HEY1*	*NTRK3*	*TEAD1*
ERBB4	***PDGFRA***	*CITED2*	*HMGA1*	*NUTM1*	*TFCP2*
FGFR1	*PIK3CA*	*CLDN18*	*HMGA2*	*OGA*	*TFE3*
FGFR2	*POLD1*	*COL1A1*	*JAZF1*	*PAX3*	*TFEB*
FGFR3	*POLE*	*CREB1*	*KIT*	*PAX7*	*TGFBR3*
FGFR4	*PRKD1*	*CREB3L1*	*MAML2*	*PBX1*	*TRIM11*
FOXL2	*PTPN11*	*CREB3L2*	*MAP3K8*	*PBX2*	*USP6*
GNA11	***PTEN***	*CREM*	*MBTD1*	*PDGFRA*	*VGLL2*
GNAQ	*RAC1*	*CSF1*	*MEIS1*	*PDGFRB*	*WT1*
GNAS	***RET***	*CTNNB1*	*MET*	*PHF1*	*YAP1*
H3F3A	***SMAD4***	*DDIT3*	*MN1*	*PLAG1*	*YWHAE*
H3F3B	***STK11***	*EGFR*	*MYB*	*PRKACA*	*ZNF444*
HIST1H3B	*TERT*	*ETV6*	*MYBL1*	*PRKACB*	
HIST1H3C	***TP53***	*EWRS1*	*NAB2*	*PRKCA*	

Table S2. Clinical, endoscopic, cytohistological, FISH and molecular data for all 127 procedures, ordered by procedure number.

PRX: procedure number X, PX: patient number X, M: male, F: female, ERCP: endoscopic retrograde cholangiopancreatography, SOC: single operator cholangioscopy.

PDF-link.

Supplementary references

1. Bournet B, Selves J, Grand D, et al. Endoscopic Ultrasound–guided Fine-Needle Aspiration Biopsy Coupled With a KRAS Mutation Assay Using Allelic Discrimination Improves the Diagnosis of Pancreatic Cancer. Journal of Clinical Gastroenterology 2015;49:50–6.
2. Syrykh C, Gorez P, Péricart S, et al. Molecular diagnosis of T-cell lymphoma: a correlative study of PCR-based T-cell clonality assessment and targeted NGS. Blood Advances 2021;5:4590–3.
3. Koeppel F, Muller E, Harlé A, et al. Standardisation of pathogenicity classification for somatic alterations in solid tumours and haematologic malignancies. European Journal of Cancer 2021;159:1–15.
4. Escudié F, Ivashchenko V, Brousset P. bialimed/SoFuR: v0.8.0 2022.
5. Bontoux C, Vigier A, Valentin T, et al. A challenging case of aggressive composite hemangioendothelioma with neuroendocrine differentiation and PTBP1::MAML2 fusion. Genes, Chromosomes and Cancer 2024;63:e2324.
