# Supplementary material for: Molecular endoscopy with next-generation sequencing improves diagnosis of cholangiocarcinoma in patients with extrahepatic biliary strictures
Source: JHEP Rep. 2026 Feb 16;8(5):101788. doi: 10.1016/j.jhepr.2026.101788 (PMC13058966; doi:10.1016/j.jhepr.2026.101788)
Supplement: Multimedia component 5 [file mmc5.pdf]

Molecular endoscopy with next-generation sequencing improves diagnosis of cholangiocarcinoma in patients with extrahepatic biliary strictures

Authors

Anne-Cécile Brunac, Adrian Culetto, Hadrien Reboul, …, Céline Basset, Jean-Marie Peron, **Janick Selves,**

Correspondence

brunac.annececile@iuct-oncopole.fr (A.-C. Brunac).

Graphical abstract

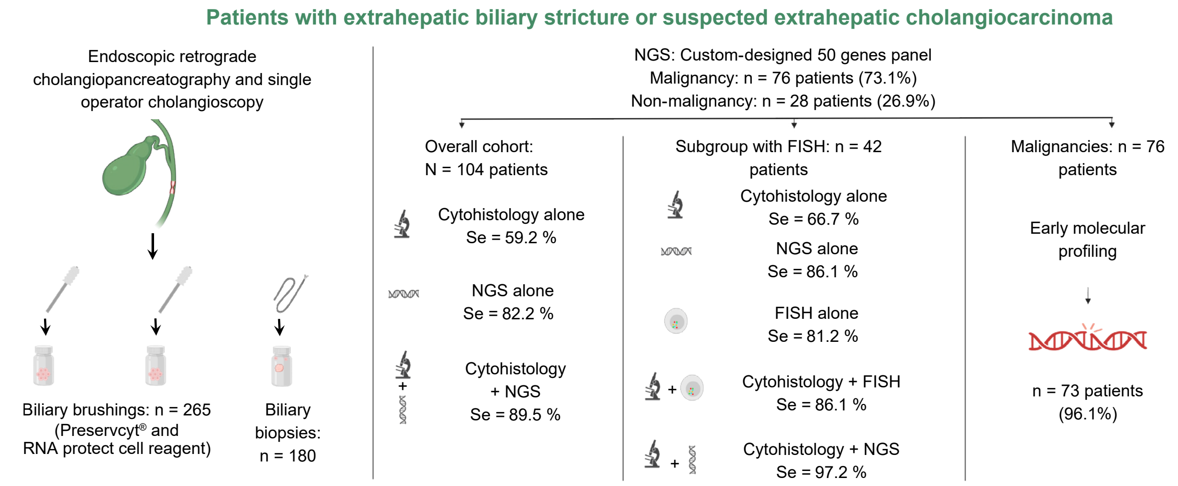

Highlights:

- Targeted NGS improves malignancy detection in extrahepatic biliary strictures.
- NGS shows higher sensitivity than cytohistology and FISH alone.
- Combining NGS and cytohistology yields the highest diagnostic performance.
- NGS enables early molecular profiling to guide therapeutic decision-making.

Impact and implications:

Differentiating benign from malignant extrahepatic biliary strictures remains a major clinical challenge, justifying the need for more sensitive and specific diagnostic tools beyond conventional cytohistology. Our findings show that combining next-generation sequencing with cytohistology significantly improves diagnostic performance, enables early molecular profiling, and is easily applicable in clinical laboratories. These results are especially relevant for patients with suspected cholangiocarcinoma and patients with primary sclerosing cholangitis, where timely and accurate diagnosis is critical and often difficult. Integrating next-generation sequencing into routine diagnostic workflows could reduce diagnostic delays and guide therapeutic decisions in patients with biliary tract lesions.

https://doi.org/10.1016/j.jhepr.2026.101788
© 2026 The Authors. Published by Elsevier B.V. on behalf of European Association for the Study of the Liver (EASL). This is an open access article under the CC BY license (http://creativecommons.org/licenses/by/4.0/).

Molecular endoscopy with next-generation sequencing improves diagnosis of cholangiocarcinoma in patients with extrahepatic biliary strictures

Anne-Cécile Brunac[1,*], Adrian Culetto[2], Hadrien Reboul[1], Karl Barange[3], Louis Buscail[2], Nadim Fares[4], Ronan Guillemin[1], Emily Alouani[4], David Grand[1], Solène Evrard[1], Céline Basset[1], **Jean-Marie Peron**[3,†], **Janick Selves**[1,†]

JHEP Reports **2026**. vol. 8 | 1–10

Check for updates

Background & Aims: Differentiating benign from malignant extrahepatic biliary strictures remains a diagnostic challenge, despite advances in sampling techniques and ancillary tests. Next-generation sequencing (NGS) may improve diagnostic performance and enable essential molecular profiling for malignancies. We aimed to assess the diagnostic performance of targeted NGS compared to cytohistology and fluorescence *in situ* hybridization (FISH), using samples exclusively collected via optimized single-operator cholangioscopy.

Methods: We prospectively enrolled 104 patients with extrahepatic biliary strictures or suspected cholangiocarcinoma. A definitive diagnosis was established through cytohistology and clinical follow-up. In total, 445 samples (265 brushings and 180 biopsies) were collected. Targeted DNA sequencing was performed using a custom-designed 50-gene panel. FISH was retrospectively performed in a subgroup of 42 patients. The diagnostic performance of all three modalities was compared.

Results: NGS achieved a sensitivity of 82.2% for malignancy, significantly higher than cytohistology alone (59.2%, $p = 1.9\text{x}10^{-3}$). Combining both modalities increased sensitivity to 89.5% ($p = 4.1\text{x}10^{-2}$). In the 42-patient subgroup, combining NGS and cytohistology achieved 97.2% sensitivity, superior to cytohistology (66.7%), FISH (81.2%), the combination of cytohistology and FISH (86.1%) and NGS alone (86.1%). Among malignant cases, NGS succeeded in 96.1%, enabling early molecular profiling for these patients.

Conclusions: The combination of cytohistology and NGS significantly improves diagnostic performance for cholangiocarcinoma in patients with extrahepatic biliary strictures using specimens obtained via single-operator cholangioscopy. This approach also enables early molecular profiling of low-cellularity specimens and may reduce diagnostic delays while optimizing therapeutic decision-making.

© 2026 The Authors. Published by Elsevier B.V. on behalf of European Association for the Study of the Liver (EASL). This is an open access article under the CC BY license (http://creativecommons.org/licenses/by/4.0/).

Introduction

Extrahepatic biliary strictures (EBS) can result from both malignant and benign causes, presenting a major diagnostic challenge. One of the main benign causes of EBS is primary sclerosing cholangitis (PSC), which is associated with an increased risk of cholangiocarcinoma (CCA), with an annual risk of 1.5-2% and a 400-fold increase compared to the general population.[1] Other benign causes of extrahepatic biliary strictures include infection-related cholangitis, IgG4-mediated cholangitis and pancreatitis, chronic pancreatitis, extrinsic compression by a pancreatic fluid collection, iatrogenic injuries, vascular conditions, and Mirizzi syndrome.[2] Malignant strictures are most commonly due to extrahepatic CCA (eCCA), pancreatic adenocarcinoma, ampullary cancer and, less frequently, gallbladder cancer, hepatocellular carcinoma and metastatic cancers.[2,3] EBS pose a major diagnostic challenge due to limited accessibility for tissue sampling and the potential for benign mimics, such as PSC, which can lead to diagnostic pitfalls.

Endoscopic retrograde cholangiopancreatography (ERCP) remains the gold standard technique, enabling collection of both biliary brushings and forceps biopsies. However, the sensitivity for detecting malignant biliary strictures is limited (45–56% for brushings and 48–67% for biopsies, despite excellent specificity of approximately 99%), and combining both modalities yields only a moderate increase in sensitivity.[4,5] Although ERCP with cholangioscopy-directed biopsies has moderately improved diagnostic performance, particularly in PSC cases, and is feasible in referral hospitals,[1] its performance remains insufficient.[6–10] This leads to patients frequently undergoing multiple procedures and delays in therapeutic decision-making. To improve the detection of

* Corresponding author. Address: Anatomie et cytologie pathologiques, 1 avenue Irène Joliot-Curie, 31059 Toulouse cedex 9, France; Tel.: +33531156203.
E-mail address: brunac.annececile@iuct-oncopole.fr (A.-C. Brunac).
† JM Peron and J Selves contributed equally to this work and share last authorship.
https://doi.org/10.1016/j.jhepr.2026.101788

malignant strictures, several ancillary techniques, most of them based on molecular alterations, can be used.[11] Although FISH has been the most evaluated method to date, it is not widely used in routine practice and its sensitivity remains limited, underscoring the need to implement new technologies.

CCAs are characterized by numerous tumor-associated gene alterations, some prevalent across all anatomical sites (*KRAS* and *TP53* mutations), others more subtype-specific (*IDH1/2* mutations and *FGFR2* fusions in intrahepatic CCA [iCCA], *ERBB2* amplifications in eCCA and gallbladder adenocarcinomas). Several of these alterations represent actionable targets, positioning molecular profiling as a key step in therapeutic management.[12,13] The utility of next-generation sequencing (NGS) for diagnosing biliary strictures has been investigated by few teams, with promising initial results.[14–16]

Building on similar approaches, the first objective of this study was to evaluate the diagnostic performance for malignancy of targeted NGS in EBS and compare it with FISH, for the first time in a prospective cohort comprising solely extrahepatic biliary samples obtained through optimized, single-operator cholangioscopy using the SpyGlass™ system. The second objective was to assess the feasibility of early molecular profiling of eCCA on both DNA and RNA.

Patients and methods

Study cohort

All consecutive patients diagnosed with EBS and/or suspected eCCA were prospectively enrolled in our tertiary center, Toulouse University Hospital, France, between November 2020 and June 2025. Samples were collected during procedures with standard ERCP and/or single-operator cholangioscopy (SOC) with the SpyGlass™ system. All patients were aged over 18 years and provided informed consent. Ethical approval for this study was obtained from Toulouse University Hospital (Approval number: 2023-065), with full compliance with ethical standards. All research was conducted in accordance with both the Declarations of Helsinki and Istanbul. Personal and medical data, including demographic, clinical, endoscopic, and follow-up information, were collected and processed for research purposes. A definitive diagnosis of malignancy was established based on histological or cytological evidence of at least high-grade dysplasia, clinical or radiological progression during follow-up, or malignancy-related death.

A specific protocol was established for collecting and processing the different samples, which is detailed in the supplementary methods and summarized in Fig. 1. We compared

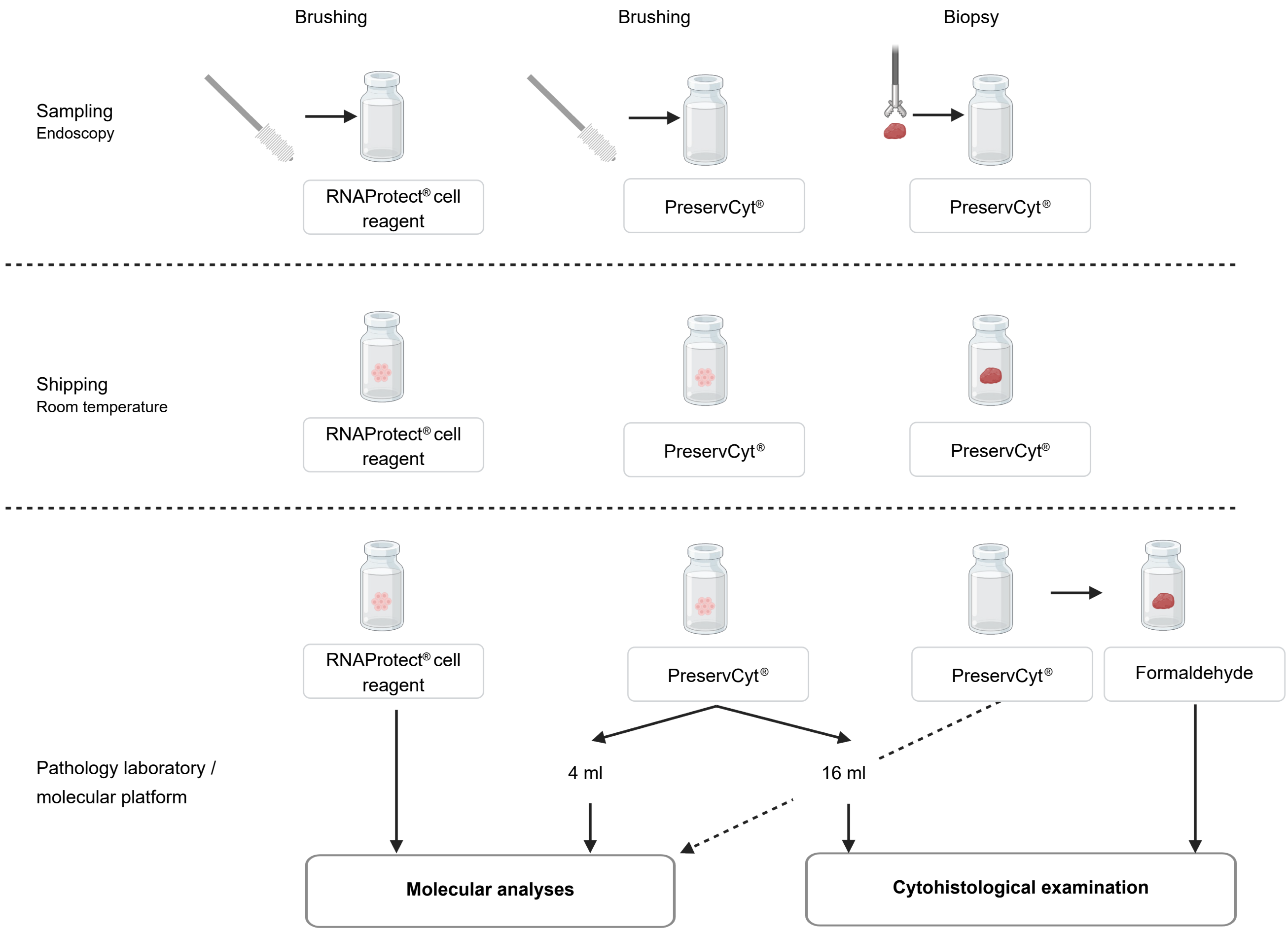

Fig. 1. Workflow protocol. The workflow protocol was established through collaboration between endoscopists, pathologists, and the molecular biology platform for the collection and processing of the extrahepatic biliary samples.

two sampling modalities: biliary brushings and biliary biopsies, and two types of preservative solutions: PreservCyt® solution (Hologic Corp., USA), commonly used in clinical laboratories, and RNAprotect® Cell Reagent (Qiagen, Germany), which preserves nucleic acids with high quality. Briefly, all samples were collected following the dedicated protocol developed collaboratively by endoscopists, pathologists, and the molecular biology platform. Whenever possible, two brushings and at least one biopsy were obtained. Upon receipt at the pathology laboratory/molecular platform, biopsies were formalin-fixed and paraffin-embedded for cytohistological examination. Cell pellets from brushings preserved in RNAProtect® Cell Reagent were used exclusively for molecular analyses, while brushings collected in PreservCyt® were divided, with 4 ml allocated to molecular testing and 16 ml processed for cytohistological evaluation (cell blocks).

Cytohistological examination

Slides from biliary brushings and biopsies were reviewed by two expert gastrointestinal pathologists (JS and ACB). Cytohistological findings were categorized using a simplified classification based on the WHO system:[17]

- Negative for malignancy: no morphological abnormality, reactive changes, lesions indeterminate for dysplasia, or low-grade dysplasia.
- Positive for malignancy: high-grade dysplasia, *in situ* carcinoma or invasive carcinoma.

DNA and RNA sequencing

All specimens including brushings in PreservCyt®, brushings in RNAProtect® Cell Reagent, and PreservCyt® biopsy supernatants, were processed uniformly. When multiple samples were available for a patient, all were processed and, if qualified, sequenced. Capture-based targeted NGS was performed on an Illumina® NextSeq 550Dx in paired-end sequencing (2x149 cycles) (San Diego, CA) using a custom-designed panel targeting 50 genes for DNA sequencing, routinely utilized in our clinical laboratory for molecular profiling of solid tumors, enabling the detection of single nucleotide variants and copy number variations of relevant genes. RNA sequencing was performed using our custom-designed panel targeting 98 genes, routinely used in our clinical laboratory. Protocols of DNA and RNA sequencing and lists of the genes are described in the supplementary methods and Table S1.

Procedures were classified based on the most significant molecular alteration detected across all available samples: "Pathogenic" if at least one pathogenic or likely pathogenic variant was identified in any sample; "VUS" if only variants of unknown significance were detected, without any pathogenic variants; and "Wild type" if no such variants were found in any sample.

Fluorescence *in situ* hybridization (FISH) analysis

Hybridization was performed on 4-µm-thick sections of biopsy specimens or cell blocks. The UroVysion® assay was employed as described previously.[18] Results were assessed by quantifying the number of signals from centromeric probes for chromosomes CEP3 (red), CEP7 (green), CEP17 (aqua), and the locus-specific identifier 9p21 (gold) in atypical cells. Specimens were classified as "positive" if the following criteria were met: presence of >2 signals for any of CEP3, CEP7, or CEP17 in ≥4 cells (or >10%), or the loss of both locus-specific identifier 9p21 signals in ≥12 cells. A minimum of 25 well-defined, clearly visualized, and non-overlapping tumor nuclei were required for the analysis. All slides were reviewed independently by two experienced gastrointestinal pathologists (ACB and JS).

HER2 status

HER2 status was evaluated by immunohistochemistry +/- *in situ* hybridization on FFPE blocks of CCAs according to the procedure usually used in the laboratory (detailed in the supplementary methods).

Statistics

Baseline characteristics were reported as counts and percentages for categorical variables and as the mean with minimum and maximum values for continuous variables. Sensitivity, specificity, positive predictive value (PPV), and negative predictive value (NPV) were calculated retrospectively for each test relative to the definitive diagnosis using standard 2 × 2 contingency tables. To calculate the diagnostic performances, we considered only the first procedure performed for each patient. In cases where a patient underwent multiple procedures, only the earliest result was included in the analysis to avoid bias due to overrepresentation of patients with multiple procedures. Comparisons of sensitivity and specificity between tests were performed using the exact McNemar test, while PPV and NPV were compared as described by Moskowitz and Pepe.[19] Statistical significance was defined as a *p*-value <0.05. All analyses were conducted using R statistical software (version 4.4).

Results

Cohort description

The study included 104 patients who underwent 127 procedures (15 patients with >1 procedure), all presenting with EBS and/or suspected eCCA diagnosed by MRI. Comprehensive clinical and endoscopic characteristics for the 104 patients are summarized in Table 1. Comprehensive clinical, endoscopic, sampling, pathological, therapeutic and follow-up data for each procedure are detailed in Table S2.

In total, 84 patients (80.8%) presented clinical symptoms (including jaundice, abdominal pain, pruritus, alteration of general health condition, fever, nausea), and five patients (4.8%) presented cholestasis without symptoms. A total of 11 patients (10.6%) were included in the context of a PSC follow-up. The mean follow-up duration was 14.8 months (95% CI 12.3-17.2).

Definitive diagnoses identified malignant causes in 76 patients (73.1%) and non-malignant causes in 28 patients (26.9%). Definitive diagnosis of malignancy was established based on cytohistological examination (from ERCP, metastasis biopsy or surgical resection), clinical evolution, imaging, and/or endoscopic findings (Fig. 2, Table S2). In non-malignant cases, definitive diagnosis relied primarily on clinical evolution, either alone or in combination with additional diagnostic methods,

Table 1. Clinical and endoscopic characteristics for 104 patients.

Clinicopathological characteristics of all patients (N = 104)	
Gender, n (%)	
Female	34 (32.7)
Male	70 (67.3)
Mean age, years (range)	66.7 (24-87)
Repeat procedure, n (%)	
Yes	15 (14.4)
No	89 (85.6)
Endoscopic lesion, n (%)	
Stricture	78 (75)
Mass	6 (5.8)
Stricture and mass	17 (16.3)
Inflammatory	3 (2.9)
Location of lesion, n (%)	
Perihilar	50 (48.1)
Perihilar and distal	7 (6.7)
Distal	47 (45.2)
Definitive diagnosis, n (%)	
Malignant causes	76 (73.1)
Cholangiocarcinoma	55 (52.9)
Pancreatic adenocarcinoma	8 (7.7)
Ampullary adenoma with high-grade dysplasia/ adenocarcinoma	3 (2.9)
Gallbladder adenocarcinoma	3 (2.9)
Intraductal papillary neoplasm of the bile duct with high-grade dysplasia	2 (1.9)
Biliary high-grade dysplasia (on primary sclerosing cholangitis)	1 (1)
Duodenal adenocarcinoma	1 (1)
Hepatocellular carcinoma	1 (1)
Neuroendocrine carcinoma of the main bile duct	1 (1)
Extrinsic compression by carcinomatosis	1 (1)
Non-malignant causes	28 (26.9)
Primary sclerosing cholangitis/overlap syndrome	11 (10.6)
Benign stricture (infectious, iatrogenic, post-acute pancreatitis, post-traumatic)	10 (9.6)
Lithiasis	3 (2.9)
IgG4-related cholangitis	2 (1.9)
Secondary biliary cirrhosis	1 (1)
Mirizzi syndrome	1 (1)

including cytohistological examination, imaging, endoscopy, biological analyses, constitutional genetics, and exploratory surgery (Fig. 2, Table S2). The mean time from the first sampling procedure to definitive diagnosis for the overall cohort was 2.7 months (range 0–45.3). This interval was shorter in patients with malignant cause (mean 1.9 months, range 0–45.3) compared with those with non-malignant cause (mean 5.1 months, range 0–24). Fig. S1 depicts the initial lesion/ stricture classification (suspect, indeterminate, not suspect), the time from the first sampling procedure to definitive diagnosis, as well as the duration of follow-up, for all patients.

Among patients with a definitive diagnosis of malignancy, clinical management was guided by multidisciplinary discussion and included surgical resection, with or without adjuvant chemotherapy, radiochemotherapy, systemic therapy (chemotherapy with or without immunotherapy), or best supportive care, as appropriate (Table S2). All patients who underwent surgical resection (n = 20) had malignancy confirmed on the resection specimen.

In total, 445 samples were collected, with 265 biliary brushings and 180 biliary biopsies, including 165 biopsies obtained using the SpyBite™ forceps (Fig. S2).

Cytohistological examination

Samples collected for cytohistological evaluation from the 127 procedures included biliary brushings alone in 22 procedures (17.3%), biliary biopsies alone in six procedures (4.7%), and both biliary brushings and biopsies in 99 procedures (78%). Cytohistological analysis revealed: nine procedures (7.1%) without morphological abnormalities, 37 procedures (29.1%) with reactive changes, 18 procedures (14.2%) with lesions indeterminate for dysplasia, 11 procedures (8.6%) with low-grade dysplasia, 17 procedures (13.4%) with high-grade dysplasia or *in situ* carcinoma, and 35 procedures (27.6%) with invasive carcinoma. Representative images for each category are available in Fig. 3.

For the 90 procedures with a definitive diagnosis of malignancy, 52 (57.8%) were positive for malignancy and 38 (42.2%) were negative for malignancy according to the cytohistological examination. All 37 procedures with a definitive diagnosis of non-malignancy were negative for malignancy according to the cytohistological examination (Fig. 2).

DNA qualification and sequencing

To determine the most suitable procedure for DNA sequencing, we evaluated the DNA quality of two types of samples, biliary brushings and biopsies, preserved in PreservCyt® and in RNAprotect® Cell Reagent. Higher DNA quality was obtained from brushings compared to biopsy supernatants ($p = 1.4\times10^{-32}$), while no difference was observed between brushing samples preserved in PreservCyt® and RNAprotect® Cell Reagent ($p = 1$) (Fig. S3). Thus, DNA from biopsy supernatants was discontinued for use after the 54th procedure (except for one procedure where only a biopsy sample was available).

The flow chart in Fig. S2 shows the number of samples, procedures, and patients throughout the workflow, including collection during ERCP, DNA quality assessment, sequencing success, and identification of pathogenic variants. In total, 279/445 samples (62.7%) from 121 procedures (95.3%) were subjected to DNA sequencing. Six procedures (4.7%), including four limited only to biopsy specimens, did not yield qualified DNA. Pathogenic variants were identified in 71 procedures (55.9%), a singular VUS was identified in one procedure (0.8%) and no mutation was detected in 49 procedures (38.6%).

The correlation between endoscopic findings, cytohistological classification, DNA qualification for sequencing, and genomic alterations, along with the definitive diagnosis for each procedure is represented in Fig. 2. For the 86 procedures with a definitive diagnosis of malignancy and available material for DNA sequencing, 70 (81.4%) were classified as pathogenic, one (1.2%) was classified as VUS and 15 (17.4%) were classified as wild type. The same targeted NGS was additionally performed on surgical specimens in 7/20 patients who underwent surgical resection with confirmation of cancer and showed concordance with the molecular alterations identified in ERCP-derived samples in six cases. In one patient (P064), no variants were detected in biliary brushing samples, whereas NGS performed on the surgical resection revealed pathogenic mutations in *KRAS*, *TP53*, *SMAD4* and *CDKN2A*. For the 35

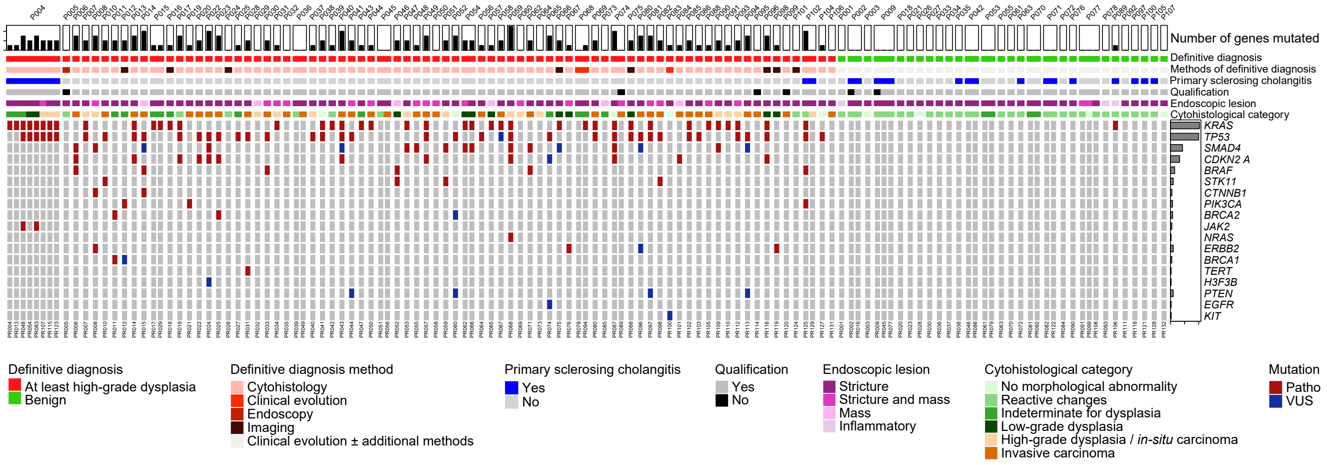

Fig. 2. Heatmap illustrating the mutational landscape of the 104 patients included in the study. Each column corresponds to a single ERCP procedure (procedure identifiers shown at the bottom), ordered according to the definitive diagnosis, and columns are grouped by patient (patient identifiers shown at the top). The heatmap displays detected pathogenic variants (dark red) and variants of unknown significance (dark blue) across the targeted genes. The bar plot above the heatmap represents the number of mutated genes identified per procedure, while the bar plot on the right shows the overall frequency of mutations for each gene across the cohort. Annotated bars above the heatmap indicate, from top to bottom: definitive diagnosis (red for at least high-grade dysplasia, green for benign); diagnostic method used to establish the definitive diagnosis (cytohistology, clinical evolution, endoscopy, imaging shown in a red gradient, and clinical evolution with or without additional methods shown in light grey); presence of primary sclerosing cholangitis (blue); DNA qualification (yes in grey, no in black); type of endoscopic lesion (stricture, stricture with mass, mass, or inflammatory lesion shown in a pink gradient); and cytohistological category (no morphological abnormality, reactive changes, indeterminate for dysplasia, low-grade dysplasia shown in a green gradient, and high-grade dysplasia/*in situ* carcinoma, invasive carcinoma shown in an orange gradient). ERCP, endoscopic retrograde cholangiopancreatography; VUS, variant of unknown significance.

procedures with a definitive diagnosis of non-malignancy and available material for DNA sequencing, pathogenic *KRAS* variants were identified for one (2.9%), and 34 (97.1%) were classified as wild type (Fig. 2).

FISH analysis

A FISH analysis was performed retrospectively on a subgroup of 47 procedures from 42 patients. These procedures were selected based on cytohistological results and availability of material: one procedure without morphological abnormalities, eight with reactive changes, 11 with lesions indeterminate for dysplasia, two with low-grade dysplasia, six with high-grade dysplasia/*in situ* carcinoma, and 18 with invasive carcinoma. FISH was applied on 41 biopsy specimens and six cell blocks. Among these, 41 procedures (87.2%) had a definitive diagnosis of malignancy, while six procedures (12.8%) were diagnosed with non-malignant conditions.

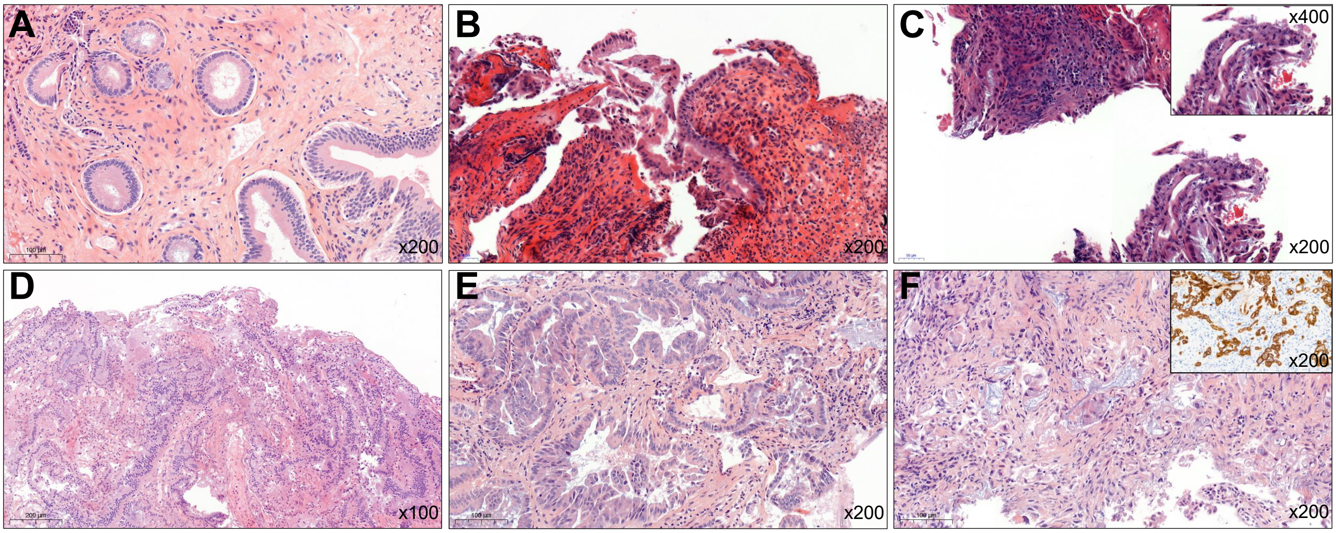

Fig. 3. Representative examples of each cytohistological diagnostic category (A) No morphological abnormalities (H&E stain, x200); (B) Reactive changes with acute inflammation (H&E, x200); (C) Lesion indeterminate for dysplasia in an inflammatory context (H&E, x200 and inset: x400); (D) Intraductal papillary neoplasm with low-grade dysplasia (H&E, x200); (E) Biliary high-grade dysplasia (H&E, x200); (F) Invasive cholangiocarcinoma (H&E, x200 and inset: cytokeratin 7 immunohistochemistry, x200).

FISH was positive for 31 procedures: 23 with polysomy (47.9%), including five with concurrent 9p21 loss (Fig. S4B and C), seven with trisomy 3 (16.7%), including two with concurrent 9p21 loss, and one with trisomy 7 (2.1%). Eight procedures showed no significant anomalies (16.7%) (Fig. S4A), and eight were uninterpretable (16.7%): four due to excessive nuclear overlap, three due to insufficient cellular material (<25 cells), and one due to hybridization failure. Detailed results for each procedure are provided in Table S2.

Comparison of the diagnostic performances of cytohistology, NGS and FISH

All results of the diagnostic performances of each diagnostic modalities for the overall cohort and the subgroup with FISH analysis are summarized in Table 2.

In the overall cohort (n = 104 patients), the diagnostic performances of NGS were significantly better than cytohistological examination with a better sensitivity for detecting at least high-grade dysplasia, a high specificity ($p = 1.9 \times 10^{-3}$), a high PPV ($p = 0.3$) and a better NPV ($p = 9.5 \times 10^{-5}$). The combination of NGS and cytohistological assessment demonstrated even better performances with a better sensitivity, similar Sp ($p = 4.1 \times 10^{-2}$) and PPV ($p = 0.4$) and a better NPV ($p = 1.4 \times 10^{-2}$).

In the subgroup with FISH (n = 42 patients), FISH showed slightly lower sensitivity than NGS but higher than cytohistology. However, its specificity and NPV were notably lower than those of both NGS and cytohistology. In terms of overall diagnostic accuracy, NGS, and particularly the combination of NGS and cytohistology, outperformed FISH alone and the combination of cytohistology and FISH.

Subanalysis of patients with primary sclerosing cholangitis

In total, 13 patients had PSC: 11 with a prior diagnosis and 2 newly diagnosed during the study. Six of them underwent repeated procedures (2–8 per patient). They all exhibited EBS. After a mean follow-up period of 22.7 months (95% CI 12-33.4), one patient developed CCA, and one patient developed high-grade biliary dysplasia (*P102* and *P004*). The remaining 11 patients exhibited only reactive cytohistological changes. No molecular alterations were detected in this group, except in one patient (P089) in whom a pathogenic *KRAS* variant was identified in one sample (p.(Gly12Asp), with a low variant allele frequency of 2.12% (Fig. 2). All 11 patients were ultimately classified as having non-complicated PSC as the definitive diagnosis. Patient P089, for whom no histological evidence of malignancy was found, was referred for liver transplantation because of recurrent episodes of cholangitis.

One patient who developed high-grade biliary dysplasia on PSC underwent eight endoscopic procedures between December 2020 and April 2025 (*P004*). Cytohistological evaluation revealed a progression of biliary dysplasia over time on the common bile duct as shown in Fig. 4. Notably, DNA sequencing identified oncogenic alterations before cytohistological confirmation of neoplasia. A pathogenic *KRAS* mutation was detected early in the course, followed by the emergence of a pathogenic *TP53* mutation 2 years later, with increasing variant allele frequencies over successive procedures. Thus, molecular alterations preceded the cytohistological

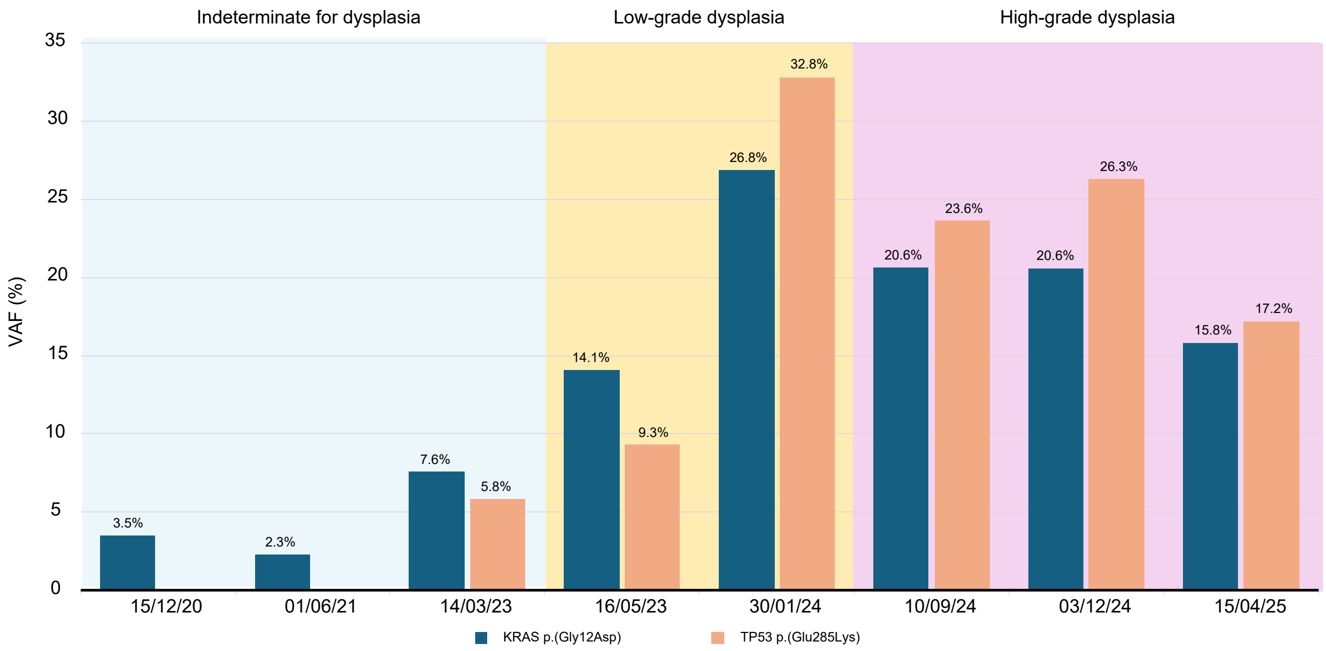

Fig. 4. Evolution of VAF over time in a patient (P004) with PSC. The plot depicts the VAF of two pathogenic variants, *KRAS* p.(Gly12Asp) (shown in blue) and *TP53* p.(Glu285Lys) (shown in orange), measured in one representative sample from each of eight ERCP procedures performed between 2020 and 2025. Each time point corresponds to a distinct procedure. Background shading indicates the cytohistological assessment at each procedure (blue: indeterminate for dysplasia; yellow: low-grade dysplasia; pink: high-grade dysplasia), illustrating the temporal relationship between molecular alterations and pathological evaluation. ERCP, endoscopic retrograde cholangiopancreatography; PSC, primary sclerosing cholangitis; VAF, variant allele frequency.

Table 2. Diagnostic performances of different diagnostic modalities.

Diagnostic modality	Se [95% CI]	Sp [95% CI]	PPV [95% CI]	NPV [95% CI]	Accuracy
**Overall cohort (N = 104 patients)**					
Cytohistology	59.2% [0.48–0.70]	100% [0.89–1.00]	100% [0.93–1.00]	47.5% [0.35–0.60]	70.2%
NGS	82.2% [0.73–0.91]	96.2% [0.89–1.00]	98.4% [0.95–1.00]	65.8% [0.51–0.81]	85.9%
Cytohistology + NGS	89.5% [0.83–0.96]	96.4% [0.90–1.00]	98.6% [0.96–1.00]	77.1% [0.63–0.91]	91.3%
**Subgroup (n = 42 patients)**					
Cytohistology	66.7% [0.51–0.82]	100% [0.50–1.00]	100% [0.88–1.00]	33.3% [0.12–0.55]	71.4%
NGS	86.1% [0.75–0.97]	100% [0.50–1.00]	100% [0.90–1.00]	54.5% [0.25–0.84]	88.1%
Cytohistology + NGS	97.2% [0.92–1.00]	100% [0.50–1.00]	100% [0.91–1.00]	85.7% [0.60–1.00]	97.6%
FISH	81.2% [0.68–0.95]	50% [0.01–0.99]	92.9% [0.83–1.00]	25% [0.00–0.55]	77.8%
Cytohistology + FISH	86.1% [0.75–0.97]	66.7% [0.29–1.00]	93.9% [0.86–1.00]	44.4% [0.12–0.77]	83.3%

FISH, fluorescence *in situ* hybridization; NGS, next-generation sequencing; NPV, negative predictive value; PPV, positive predictive value; Se, sensitivity; Sp, specificity. Se, Sp, PPV, and NPV were calculated retrospectively for each test relative to the definitive diagnosis using standard 2×2 contingency tables.

confirmation of neoplasia by more than 4 years. Following multidisciplinary team discussion, the patient was referred for liver transplantation.

DNA molecular profiling of malignancies

A total of 76 patients had a definitive diagnosis of malignancy, including 55 CCA, eight pancreatic adenocarcinomas (ADK), three ampullary adenoma with high-grade dysplasia/ADK, three gallbladder ADK, two intraductal papillary neoplasm of the bile duct with high-grade dysplasia, one biliary high-grade dysplasia (on PSC), one duodenal ADK, one hepatocellular carcinoma, one neuroendocrine carcinoma of the main bile duct, and one extrinsic compression by carcinomatosis (Table 1). DNA molecular profiling was successfully performed for 73 patients (96.1%).

Among the biliary cancers (CCA, gallbladder ADK, intraductal papillary neoplasm of the bile duct with high-grade dysplasia and biliary high-grade dysplasia developed on PSC), three CCA (4.9%) did not yield qualified DNA and 47 (77%) harbored at least one pathogenic variant (Fig. 2). The most common alterations included mutations in *TP53* (n = 27), *KRAS* (n = 23), *SMAD4* (n = 12), and *CDKN2A* (n = 11). Less frequent pathogenic alterations included mutations in *BRAF* (n = 4), including one targetable $BRAF^{V600E}$ mutation, *ERBB2* (n = 4), *BRCA2* (n = 2), *STK11* (n = 2), *PIK3CA* (n = 2), *BRCA1* (n = 1), *CTNNB1* (n = 1), and *NRAS* (n = 1).

Copy number analysis was retrospectively performed in 27 patients with a definitive diagnosis of malignancy, including 21 CCA, two pancreatic ADK, one gallbladder ADK, one intraductal papillary neoplasm of the bile duct with high-grade dysplasia, one biliary high-grade dysplasia (on PSC), and one case of carcinomatosis. Copy number alterations were identified in two patients. One patient with CCA (P059) exhibited *ERBB2* amplification (7–8 copies) in both analyzed samples (one brushing preserved in PreservCyt® and one in RNAprotect® Cell Reagent). Immunohistochemistry showed HER2 2+ expression, and *in situ* hybridization confirmed true amplification. Another patient with gallbladder ADK exhibited *KRAS* amplification (7 copies) in both analyzed samples, of unknown clinical significance.

To further assess HER2 status, immunohistochemistry with or without *in situ* hybridization was performed when sufficient material was available. Among 55 patients with CCA and three with gallbladder ADK, HER2 status was assessable in 38 patients. Except for patient P059, none demonstrated HER2 positivity (23 scored 0, four scored 1+, and 10 scored 2+ without amplification by *in situ* hybridization).

Exploratory RNA sequencing analysis

To evaluate the feasibility of RNA molecular profiling, a subset of 16 procedures was selected retrospectively based on a definitive diagnosis of malignancy and availability of material (14 CCA, 1 gallbladder ADK, and 1 pancreatic ADK). RNA fusion analysis was performed on 28 samples derived from these 16 procedures. One *EGFR* (ENSG00000146648) rearrangement of unknown significance (*7:55151362::7:54603740*) was identified in a CCA sample and subsequently confirmed in the corresponding surgical specimen. No fusion transcripts were detected in 12 samples, while 15 samples (53.6%) were non-contributory due to insufficient RNA quality or quantity based on our quality control metrics.

Discussion

EBS pose a significant diagnostic challenge due to the difficulty in obtaining adequate tissue samples for pathological assessment, the broad spectrum of differential diagnoses, including both malignant and benign etiologies, and the urgent need for timely management in cases of CCA. Despite advances in endoscopic and cytohistological techniques, diagnostic performances remain insufficient[7,11,20] and ERCP with cholangioscopy-directed biopsies has only moderately improved these diagnostic performances, with sensitivity rates between 60.1% and 90% depending on the generation of the system. Notably, in PSC cases, SOC has achieved sensitivities of 33–65%.[6–10]

In this prospective study, we evaluated the contribution of targeted NGS to the diagnosis of EBS using samples obtained via ERCP and SOC with the SpyGlass™ system in patients with indeterminate EBS or suspected eCCA. To the best of our knowledge, this is the first study to focus exclusively on extrahepatic samples obtained through an optimized procedure using the SpyGlass™ system and evaluating the integration of NGS to this procedure. Our findings demonstrate that combining cytohistological examination with targeted DNA sequencing improves diagnostic accuracy, reaching up to 91.3%. This outperforms cytohistology alone (70.2%) and targeted NGS alone (85.9%). Moreover, our results surpass those reported in previous studies, where the sensitivity of NGS alone ranged from 73% to 75%, compared to 82.2% in our study, and the combined sensitivity of NGS and

cytohistology reached 89.5%, exceeding the previously reported range of 83% to 85.7%.[14,16,21] Additionally, our method gives better results obtained with less material waste, thus limiting invasive procedures (multiple sampling) and preserving cytological and histological material for other analyses for diagnostic or theranostic purposes. With our protocol, we showed that only a few milliliters of the preservative solution used for brushings are sufficient for both DNA and RNA sequencing. As brushings are typically performed during the initial procedure, our results indicate that additional, more invasive procedures, particularly dedicated biopsies for molecular analysis, are unnecessary. Furthermore, using brushings rather than biopsies may reduce the risk of false negatives due to intratumoral heterogeneity, as the sample is more likely to contain genetic material shed by tumor cells along the biliary tract. With our optimized procedure, only six procedures (4.7%) were not contributive for DNA sequencing. Furthermore, we observed equal performances for both preservative solutions, PreservCyt® and RNAprotect® Cell Reagent.

In addition, we explored the feasibility of targeted RNA sequencing from malignant samples obtained via ERCP and SOC using the SpyGlass™ system. Although RNA sequencing was technically successful in a subset of samples and enabled the identification of a fusion transcript in one case, the overall contribution of RNA molecular profiling in this study was limited. More than half of the samples were non-contributory (53.6%) due to insufficient RNA quality or quantity, regardless of the preservative solution used. These findings highlight the current technical challenges associated with RNA-based analyses in this context and indicate that further optimization of the method is still required.

We next compared the diagnostic performance of multicolor FISH using the UroVysion® assay to cytohistological examination and NGS. FISH has emerged as a promising tool to aid in the diagnosis of indeterminate biliary strictures, with sensitivity up to 61% but with disparate results.[11,18,22] However, it remains infrequently used in routine clinical practice and is not currently recommended.[1] FISH analysis was performed on a subgroup of 42 patients. Although it showed good sensitivity (81.2%), its specificity (50%) and NPV (25%) were low. Overall accuracy was moderate (77.8%), lower than that of NGS alone (88.1%) and markedly lower than the combination of cytohistology and NGS (97.6%). In addition, 16.7% of cases were uninterpretable. Multicolor FISH remains technically challenging and labor-intensive. As previously reported, we encountered interpretation difficulties due to frequent nuclear overlap, particularly in inflammatory contexts, which complicated probe signal enumeration. Although FISH is often considered a more affordable alternative to NGS, the growing accessibility of NGS in routine clinical practice and the progressive reduction of its cost is challenging this assumption. While NGS remains costly, its use at the time of diagnosis enhances diagnostic accuracy, potentially reducing the need for repeat procedures. Additionally, earlier diagnosis could facilitate timely therapeutic interventions and help avoid unnecessary healthcare costs associated with diagnostic delays, ultimately offering a favorable cost-benefit balance.

A major diagnostic challenge is PSC, and particularly biliary neoplasia developed on PSC, as the risk of malignancy is high and cytohistological diagnosis difficult due to important inflammation. Moreover, radical surgery or liver transplantation are the only curative treatment options but must be performed at an early stage. Three major studies focusing on patients with PSC showed the clinical utility of NGS in this context, with improved sensitivities of up to 75%, which were further improved by the combination with morphological analysis.[23–25] In our study, 13 patients (12.5%) had PSC, and molecular alterations were detected in both patients with a definitive diagnosis of high-grade dysplasia/CCA. Our findings suggest that molecular profiling may allow for the detection of early oncogenic alterations in PSC-associated biliary strictures, even when cytohistological analysis remains non-diagnostic, with potential early curative treatment. However, these results must be interpreted with caution, as the PSC subgroup was small (n = 13), limiting the strength of our conclusions. Confirmation in larger, prospective PSC cohorts will be required.

Implementing NGS at the time of diagnosis not only improves diagnostic accuracy but also enables rapid molecular profiling, allowing for the identification of therapeutic targets in a single procedure. NGS is a highly sensitive technology capable of performing multigene analysis, making it particularly suitable for these low-cellularity samples, especially given that theses samples are often the only material available as fewer than 35% of eCCA are eligible for surgical resection.[26] In addition, molecular profiling of CCA using FFPE biopsies is often challenging, with reported sequencing failure rates of up to 25%, and even higher rates observed for gene fusion detection.[27] While data on NGS of circulating tumor DNA in eCCA is limited, available studies report detection rates of genomic alterations in approximately 55% of cases.[28]

In our study, DNA sequencing was successfully performed for 96.1% of malignancies (CCA or other carcinomas), far exceeding the sequencing success rates from other types of material. Although our panel was not specifically developed for CCA (pan-tumor panel of 50 genes), it includes genes of interest for CCA treatment such as *IDH1*, *BRAF* and *ERBB2*. In addition, we explored the feasibility of molecular profiling using RNA extracted from these samples with a custom-designed targeted panel and demonstrated its applicability, supporting its potential as a screening tool for personalized therapeutic approaches.[29]

One major limitation of NGS is the risk of false positives and over-diagnosis, potentially leading to inappropriate treatment. Oncogenesis, particularly in the context of PSC, remains poorly understood.[30–32] The sequence of molecular alterations involved in malignant transformation, and their correlation with morphological features, are not yet clearly defined. *KRAS* mutations appear to be an early event in neoplastic progression, but are insufficient on their own to drive malignant transformation.[33,34] Moreover, *KRAS* mutations can also be detected in non-malignant conditions such as autoimmune pancreatitis.[35] In contrast, *TP53* alterations seem to be a late event, associated with malignant transformation.[34] Therefore, the identification of a single molecular alteration, particularly a *KRAS* mutation, is not sufficient to establish a diagnosis of malignancy. Correlation with morphological and clinical data remains essential. Similarly, the detection of molecular alterations at low allele frequencies, such as *KRAS* mutations, must be interpreted with caution, particularly when using highly sensitive NGS techniques. This concern is especially relevant in the context of cell-free DNA sequencing. Recent studies demonstrated the diagnostic potential of cell-free DNA in bile,

given the ease of bile collection during ERCP, including in the context of PSC. While this approach showed excellent sensitivity (96.4%), its specificity was lower than that observed in our study (69.2%). This was attributable to the detection of mutations in patients ultimately diagnosed with benign disease.[36,37] This may reflect true false-positive results or the detection of precancerous or early-stage malignant lesions, which will require further investigation. Other biomarkers, such as aberrant DNA methylation, seem promising,[38–40] but these technologies are not yet recommended for routine clinical practice.[1]

Our study has several limitations. First, the targeted panel used was not specifically designed for eCCA and lacked several relevant genes of interest (*ELF3*, *ARID1B*, *PBRM1*, *BAP1*), which may have led to false-negative results and affected diagnostic sensitivity. On the other hand, a smaller panel is more accessible to most laboratories and therefore more readily applicable. Moreover, although we were able to retrospectively incorporate copy number variation analysis in a subset of patients, this approach was not applicable to the entire cohort and identified only one clinically actionable *ERBB2* amplification. Consequently, the absence of systematic copy number analysis in all patients remains an important limitation. Second, the number of patients followed for strictures in the context of PSC was insufficient to accurately characterize the sequence of molecular alterations and assess their true diagnostic value. Third, the complexity and potential risk of the procedure raise practical questions, particularly regarding whether an additional brushing dedicated solely to molecular analyses should be routinely performed. Fourth, while our approach demonstrated strong diagnostic performance, it was conducted using the SpyGlass^{TM} system, which is not commonly employed as a first-line diagnostic tool. However, the EASL guidelines acknowledge its use in the initial ERCP can be cost effective in expert centers.[1] Fifth, although technically feasible, RNA sequencing showed a high failure rate and limited added diagnostic or therapeutic value in this study. Given the low proportion of contributory samples and the small number of patients analyzed, RNA sequencing from ERCP-derived material cannot currently be recommended for routine clinical use (avoiding waste of material) and will require further methodological optimization. This is notably an important limitation as *FGFR2* fusions, a rare but important therapeutic target in eCCA, could not be analyzed in our study. Finally, the actual impact on therapeutic decision-making, potential time-saving in diagnosis, and cost-effectiveness of this strategy remain to be determined.

In conclusion, our findings support the clinical value of integrating NGS with cytohistological evaluation of extrahepatic biliary specimens obtained through SOC. This combined approach not only improves diagnostic performances but also facilitates the early identification of targetable genomic alterations at the time of diagnosis, therefore optimizing therapeutic decision-making. An implication of these results is the need to reconsider and potentially redefine surveillance strategies for EBS, particularly for patients lacking morphological evidence of malignancy but harboring pathogenic variants.

Affiliations

[1]*Department of Pathology, Institut Universitaire du Cancer de Toulouse - Oncopole, Toulouse University Hospital, 1 avenue Irène Joliot-Curie, 31059 Toulouse cedex 9, France;* [2]*Department of Gastroenterology and Pancreatology, Toulouse University Hospital, 1 avenue Jean Poulhès, TSA 50032, Toulouse Cedex 9, 31059, France;* [3]*Department of Hepatology, Toulouse University Hospital, 1 avenue Jean Poulhès, TSA 50032, Toulouse Cedex 9, 31059, France;* [4]*Department of Digestive Oncology, Toulouse University Hospital, 1 avenue Jean Poulhès, TSA 50032, Toulouse Cedex 9, 31059, France*

Abbreviations

ADK, adenocarcinoma; CCA, cholangiocarcinoma; cfDNA, cell-free DNA; EBS, extrahepatic biliary strictures; eCCA, extrahepatic cholangiocarcinoma; ERCP, endoscopic retrograde cholangiopancreatography; FISH, fluorescence *in situ* hybridization; iCCA, intrahepatic cholangiocarcinoma; NGS, next-generation sequencing; NPV, negative predictive value; PPV, positive predictive value; PSC, primary sclerosing cholangitis; SOC, single-operator cholangioscopy; VUS, variant of unknown significance.

Financial support

No financial support was received to produce this manuscript.

Conflict of interest

Nothing to report for all authors.
Please refer to the accompanying ICMJE disclosure forms for further details.

Authors' contributions

JS, JMP, CB and ACB designed the study. AC, KB, LB and JMP performed endoscopic procedures. JS and ACB performed cytohistological examination. ACB and JS performed FISH analyses. ACB, JS, HR, DG, RG and SE performed molecular analyses. AC, KB, LB, JMP, NF and EA followed the patients. ACB collected the data. ACB and HR performed statistical analyses. ACB, JMP and JS wrote the article. All authors reviewed the article.

Declaration of generative AI and AI-assisted technologies in the writing process

During the preparation of this work the authors used ChatGPT in order to improve language and readability only. After using this service, the authors reviewed and edited the content as needed and take full responsibility for the content of the publication.

Data availability

The authors declare that the data presented in this article are available as raw data (raw data files and protocols) upon request to the corresponding author.

Supplementary data

Supplementary data to this article can be found online at https://doi.org/10.1016/j.jhepr.2026.101788.

References

Author names in bold designate shared co-first authorship

[1] Marzioni M, Maroni L, Aabakken L, et al. European Association for the Study of the Liver. EASL Clinical Practice Guidelines on the management of extrahepatic cholangiocarcinoma. J Hepatol 2025;83:211–238.
[2] Elmunzer BJ, Maranki JL, Gómez V, et al. ACG clinical guideline: diagnosis and management of biliary strictures. Am J Gastroenterol 2023;118:405–426.
[3] Bray F, Laversanne M, Sung H, et al. Global cancer statistics 2022: GLOBOCAN estimates of incidence and mortality worldwide for 36 cancers in 185 countries. CA: A Cancer J Clinicians 2024;74:229–263.
[4] Navaneethan U. Comparative effectiveness of biliary brush cytology and intraductal biopsy for detection of malignant biliary strictures: a systematic review and meta-analysis. Gastrointest Endosc 2015;81:168–176.
[5] Yoon SB, Moon S-H, Ko SW, et al. Brush cytology, forceps biopsy, or endoscopic ultrasound-guided sampling for diagnosis of bile duct cancer: a meta-analysis. Dig Dis Sci 2022;67:3284–3297.

[6] Navaneethan U, Hasan MK, Lourdusamy V, et al. Single-operator cholangioscopy and targeted biopsies in the diagnosis of indeterminate biliary strictures: a systematic review. Gastrointest Endosc 2015;82:608–614.e2.
[7] Laleman W, Verraes K, Van Steenbergen W, et al. Usefulness of the single-operator cholangioscopy system SpyGlass in biliary disease: a single-center prospective cohort study and aggregated review. Surg Endosc 2017;31:2223–2232.
[8] Navaneethan U, Moon JH, Itoi T. Biliary interventions using single-operator cholangioscopy. Dig Endosc 2019;31:517–526.
[9] Wen L-J, Chen J-H, Xu H-J, et al. Efficacy and safety of digital single-operator cholangioscopy in the diagnosis of indeterminate biliary strictures by targeted biopsies: a systematic review and meta-analysis. Diagnostics 2020;10:666.
[10] **Gerges C**, **Beyna T**, Tang RSY, et al. Digital single-operator peroral cholangioscopy-guided biopsy sampling versus ERCP-guided brushing for indeterminate biliary strictures: a prospective, randomized, multicenter trial (with video). Gastrointest Endosc 2020;91:1105–1113.
[11] Layfield L. Role of ancillary techniques in biliary cytopathology specimens. Acta Cytol 2020;64:175–181.
[12] **Jusakul A**, **Cutcutache I**, **Yong CH**, et al. Whole-genome and epigenomic landscapes of etiologically distinct subtypes of cholangiocarcinoma. Cancer Discov 2017;7:1116–1135.
[13] Nakamura H, Arai Y, Totoki Y, et al. Genomic spectra of biliary tract cancer. Nat Genet 2015;47:1003–1010.
[14] Dudley JC, Zheng Z, McDonald T, et al. Next-generation sequencing and fluorescence in situ hybridization have comparable performance characteristics in the analysis of pancreaticobiliary brushings for malignancy. J Mol Diagn 2016;18:124–130.
[15] Bankov K, Döring C, Schneider M, et al. Sequencing of intraductal biopsies is feasible and potentially impacts clinical management of patients with indeterminate biliary stricture and cholangiocarcinoma: clinical and Translational Gastroenterology, vol. 9; 2018, e151.
[16] Singhi AD, Nikiforova MN, Chennat J, et al. Integrating next-generation sequencing to endoscopic retrograde cholangiopancreatography (ERCP)-obtained biliary specimens improves the detection and management of patients with malignant bile duct strictures. Gut 2020;69:52–61.
[17] Pitman MB, Centeno BA, Reid MD, et al. The world health organization reporting system for pancreaticobiliary cytopathology. Acta Cytol 2022;67:304–320.
[18] Ainthachot S, Sa-ngiamwibool P, Thanee M, et al. Chromosomal aberrations, visualized using UroVysion® fluorescence in-situ hybridization assay, can predict poor prognosis in formalin-fixed paraffin-embedded tissues of cholangiocarcinoma patients. Hum Pathol 2022;126:31–44.
[19] Moskowitz CS, Pepe MS. Comparing the predictive values of diagnostic tests: sample size and analysis for paired study designs. Clin Trials 2006;3:272–279.
[20] Bang JY, Navaneethan U, Hasan M, et al. Optimizing outcomes of single-operator cholangioscopy–guided biopsies based on a randomized trial. Clin Gastroenterol Hepatol 2020;18:441–448.e1.
[21] Bardhi O, Jones A, Ellis D, et al. Next-generation sequencing improves the detection of malignant biliary strictures and changes management. Gastrointest Endosc 2024:S0016510724038288.
[22] Ahsan BU, Jin M, Theisen BK, et al. Comparison of fluorescence in situ hybridization and cytology for the accurate detection of malignant biliary strictures, with emphasis on unusual results. J Am Soc Cytopathology 2025:S2213294525000456.
[23] Scheid JF, Rosenbaum MW, Przybyszewski EM, et al. Next-generation sequencing in the evaluation of biliary strictures in patients with primary sclerosing cholangitis. Cancer Cytopathology 2022;130:215–230.
[24] Kamp EJCA, Dinjens WNM, Van Velthuysen M-LF, et al. Next-generation sequencing mutation analysis on biliary brush cytology for differentiation of benign and malignant strictures in primary sclerosing cholangitis. Gastrointest Endosc 2023;97:456–465.e6.
[25] Boyd S, Mustamäki T, Sjöblom N, et al. NGS of brush cytology samples improves the detection of high-grade dysplasia and cholangiocarcinoma in patients with primary sclerosing cholangitis: a retrospective and prospective study. Hepatol Commun 2024;8.
[26] Esmail A, Badheeb M, Alnahar B, et al. Cholangiocarcinoma: the current status of surgical options including liver transplantation. Cancers 2024;16:1946.
[27] Lamarca A, Kapacee Z, Breeze M, et al. Molecular profiling in daily clinical practice: practicalities in advanced cholangiocarcinoma and other biliary tract cancers. J Clin Med 2020;9:2854.
[28] Ettrich TJ, Schwerdel D, Dolnik A, et al. Genotyping of circulating tumor DNA in cholangiocarcinoma reveals diagnostic and prognostic information. Sci Rep 2019;9:13261.
[29] Verdaguer H, Saurí T, Acosta DA, et al. ESMO scale for clinical actionability of molecular targets driving targeted treatment in patients with cholangiocarcinoma. Clin Cancer Res 2022;28:1662–1671.
[30] Karlsen TH, Folseraas T, Thorburn D, et al. Primary sclerosing cholangitis – a comprehensive review. J Hepatol 2017;67:1298–1323.
[31] Trikudanathan G, Navaneethan U, Njei B, et al. Diagnostic yield of bile duct brushings for cholangiocarcinoma in primary sclerosing cholangitis: a systematic review and meta-analysis. Gastrointest Endosc 2014;79:783–789.
[32] Kamp EJ, Dinjens WN, Doukas M, et al. Genetic alterations during the neoplastic cascade towards cholangiocarcinoma in primary sclerosing cholangitis. J Pathol 2022;258:227–235.
[33] Kubicka S, Kühnel F, Flemming P, et al. K-ras mutations in the bile of patients with primary sclerosing cholangitis. Gut 2001;48:403–408.
[34] Hsu M, Sasaki M, Igarashi S, et al. KRAS and GNAS mutations and p53 overexpression in biliary intraepithelial neoplasia and intrahepatic cholangiocarcinomas. Cancer 2013;119:1669–1674.
[35] Kamisawa T, Tsuruta K, Okamoto A, et al. Frequent and significant K-ras mutation in the pancreas, the bile duct, and the gallbladder in autoimmune pancreatitis. Pancreas 2009;38:890.
[36] Arechederra M, Rullán M, Amat I, et al. Next-generation sequencing of bile cell-free DNA for the early detection of patients with malignant biliary strictures. Gut 2022;71:1141–1151.
[37] Arechederra M, Bik E, Rojo C, et al. Mutational analysis of bile cell-free DNA in primary sclerosing cholangitis: a pilot study. Liver Int 2025;45:e70049.
[38] Vedeld HM, Folseraas T, Lind GE. Detecting cholangiocarcinoma in patients with primary sclerosing cholangitis – the promise of DNA methylation and molecular biomarkers. JHEP Rep 2020;2:100143.
[39] Vedeld HM, Grimsrud MM, Andresen K, et al. Early and accurate detection of cholangiocarcinoma in patients with primary sclerosing cholangitis by methylation markers in bile. Hepatology 2022;75:59–73.
[40] Loi E, Zavattari C, Tommasi A, et al. HOXD8 hypermethylation as a fully sensitive and specific biomarker for biliary tract cancer detectable in tissue and bile samples. Br J Cancer 2022;126:1783–1794.

Keywords: Extrahepatic biliary stricture; ERCP; NGS; Cholangiocarcinoma; Primary sclerosing cholangitis.
Received 2 September 2025; received in revised form 9 February 2026; accepted 11 February 2026; Available online 16 February 2026

Journal of Hepatology, Volume *8*

Supplemental information

Molecular endoscopy with next-generation sequencing improves diagnosis of cholangiocarcinoma in patients with extrahepatic biliary strictures

Anne-Cécile Brunac, Adrian Culetto, Hadrien Reboul, Karl Barange, Louis Buscail, Nadim Fares, Ronan Guillemin, Emily Alouani, David Grand, Solène Evrard, Céline Basset, Jean-Marie Peron, and Janick Selves

Molecular endoscopy with next-generation sequencing improves diagnosis of cholangiocarcinoma in patients with extrahepatic biliary strictures

Anne-Cécile Brunac, Adrian Culetto, Hadrien Reboul, Karl Barange, Louis Buscail, Nadim Fares, Ronan Guillemin, Emily Alouani, David Grand, Solène Evrard, Céline Basset, Jean-Marie Peron, Janick Selves

Table of contents

Supplementary methods……………………………………………………………….2

Fig. S1…………………………………………………………………………..……...……..5

Fig. S2………………………………………………………………………...………..….6

Fig. S3…………………………………………………………………………......……7

Fig. S4…………………………………………………………………….……...…..8

Table S1………………………………………………………………….……………...9

Table S2 legend……………………………………………………………………….10

Supplementary references…………………………………………….……….….…11

Supplementary methods

Sampling and sample processing

Procedures were performed by experienced endoscopists (duodenoscope: Evis Exera III TJF-Q190V, Olympus Medical Systems Corp, Japan; cholangioscope: SpyScope™ DS II M00546610, Boston Scientific, USA). Biliary brushings (Biliary Cytology Brush FS-CB-1.5-5, Cook Medical, USA) and biopsies, including biopsies obtained with SpyBite™ forceps (SpyBite™ Max 1.2mm 286cm M00546470, Boston Scientific, Costa Rica) were collected. Concerning the two types of preservative solutions, PreservCyt® solution (Hologic Corp., USA), is commonly used for cytological analysis. RNAprotect® Cell Reagent (Qiagen, Germany) is a medium validated within the BACAP protocol (Biological and Clinical Database for Pancreatic Adenocarcinoma) (NCT02818829) (1). Two brush passes were performed per procedure. The first brush was immersed in 20 mL of PreservCyt®, and the second brush in 25 mL of RNAprotect® Cell Reagent. Brushes were agitated in their respective vials to detach cells, after which they were removed, and the vials transported to the pathology laboratory. Biopsies collected with SpyBite™ forceps were immersed in 20 mL of PreservCyt® solution.

In the laboratory, brushing samples immersed in PreservCyt® solution were divided, with 16 mL allocated for cytological analysis (cell blocks) and 4 mL reserved for molecular analyses. Samples immersed in RNAProtect® Cell Reagent were used exclusively for molecular analyses. Cell pellets were prepared within 24 hours of sample collection, aliquoted, and stored at -80°C until DNA and RNA extraction. Biopsy specimens were fixed in formaldehyde and embedded in paraffin for pathological examination. Cell pellets obtained from the supernatants were processed for molecular analyses.

DNA and RNA sequencing

Simultaneous extraction of genomic DNA and RNA was performed using the AllPrep DNA/RNA/miRNA Universal Kit (Qiagen, 80224), in accordance with the manufacturer’s protocol. Extracted DNA and RNA were quantified with Qubit Fluorometer (Thermo Fisher Scientific). DNA quality was evaluated by qPCR using an in-house protocol (2). Qualified DNA was sheared using a Covaris® ME220 system.

Library preparation was performed with the Twist Library Preparation Kit, Mechanical Fragmentation (104177) using the KAPA HiFi HotStart ReadyMix DNA polymerase (Roche sequencing, KK2602), followed by capture enrichment using NGS Target Enrichment Solutions (Twist Bioscience). Sequencing was performed on an Illumina® NextSeq 550Dx in paired-end sequencing (2x149 cycles) (San Diego, CA). Our custom-designed panel targets 50 genes (Supplementary Table 1). We target a minimum depth of 150X on each targeted nucleotide and this threshold must be achieved on at least 97% of targeted nucleotides to validate the results. Data were analyzed using an in-house pipeline. Briefly, reads were aligned on a human genome (GRCh38) with BWA mem (v0.7.17) software, and variant calling was conducted with FreeBayes (v1.2.0), Mutect2 (v4.1.4.1) and VarDict-java (1.7.0). The assay's detection limit was 2% mutant allele frequency. Variants were classified based on the classification for somatic alterations in solid tumors (3). CNV were called using CNVkit (v0.9.11) and an in-house software based on Z-scores of normalized depth and allele frequency.

RNA samples underwent rRNA depletion using the KAPA RNA HyperPrep Kit with RiboErase (HMR) (Roche sequencing, 07962274001) to generate whole transcriptome stranded libraries, indexed with the unique dual Index adapter system (Twist, Bioscience, 101308). Whole transcriptome libraries were subjected to a capture enrichment step and sequenced as described for DNA. Our custom-designed panel targets 98 genes, including *FGFR2* (Supplementary Table 1). Capture allows the genes of interest from the panel to be recovered, as well as, by overflow, the translocation zone of their partners, even if they are not themselves in the panel. Fusion transcripts were detected using STAR fusion integrated into an in-house pipeline (4,5).

HER2 status evaluation :

HER2 immunohistochemistry was performed with 4B5 monoclonal antibody (05999570001, ROCHE) and *in situ* hybridization using VENTANA HER2 Dual ISH DNA Probe (08314373001, ROCHE), both on Benchmark Ultra platform. VENTANA HER2 Dual ISH DNA Probe Cocktail contains HER2 probes and Chromosome 17 probes. Results are reported as a ratio of HER2/Chromosome 17 to determine HER2

amplification status (HER2/Chromosome 17 ratio ≥ 2.0 is amplified, while a ratio < 2.0 is non-amplified).

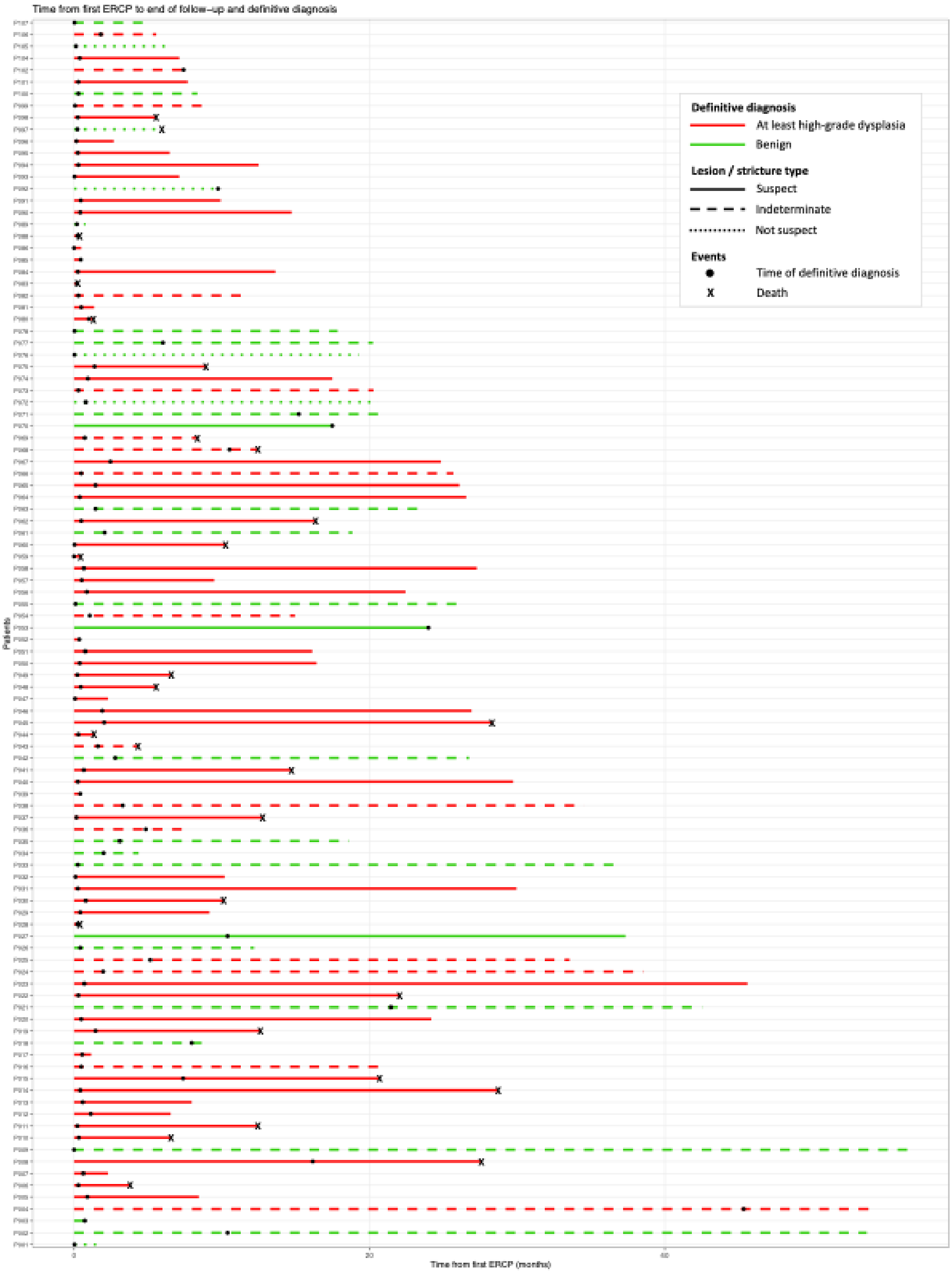

Fig. S1. Swimmer plot illustrating the time from the first sampling procedure to definitive diagnosis, along with lesion/stricture characteristics, duration of follow-up, and occurrence of death, where applicable, for all 104 patients.

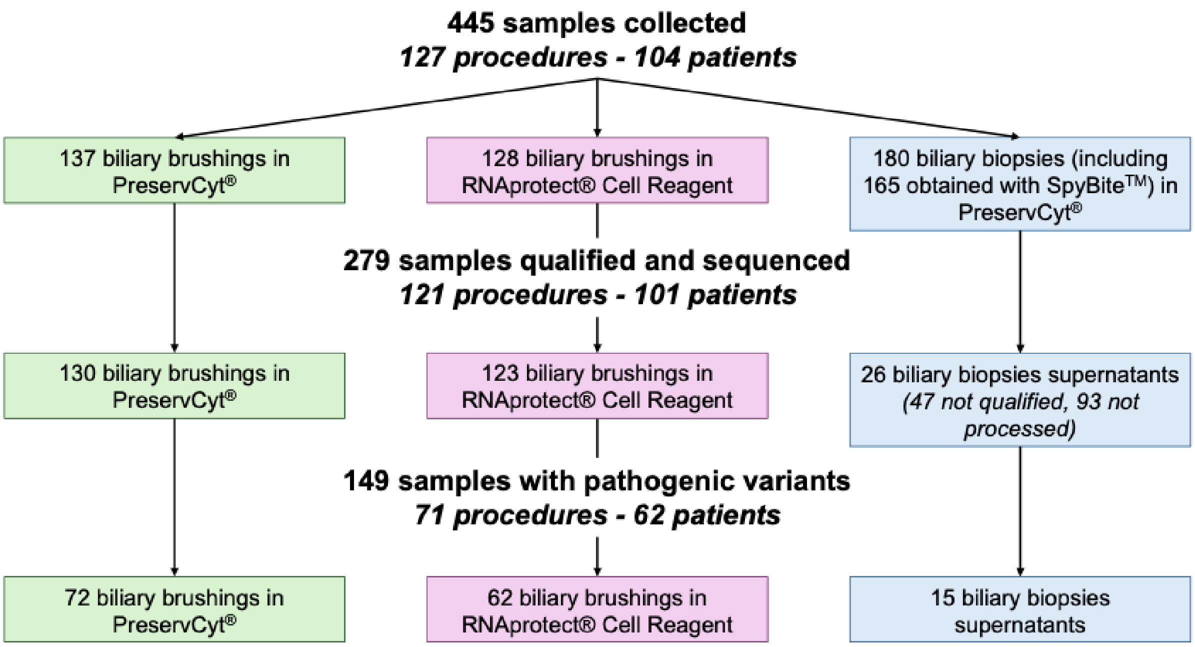

Fig. S2. Flow chart with the number of samples, procedures, and patients throughout the workflow, including collection during ERCP, DNA quality assessment, sequencing success, and identification of pathogenic variants.

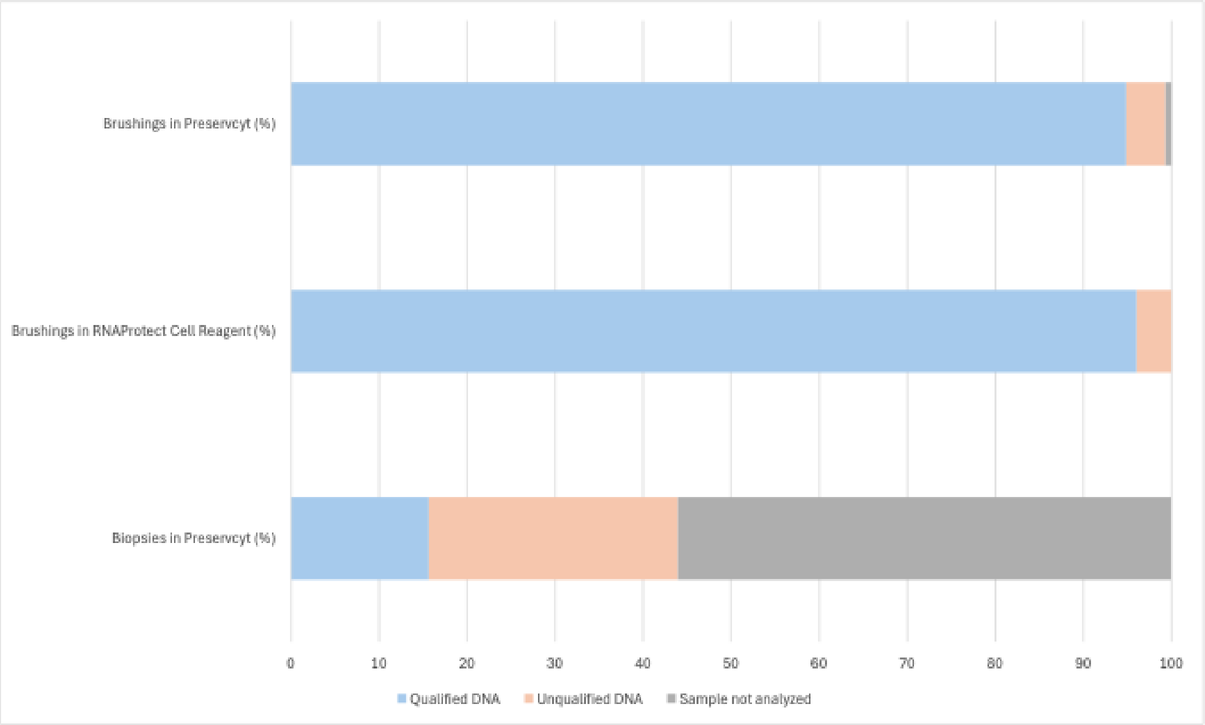

Fig. S3. DNA quality assessed by qPCR for the 3 types of samples. The quality of the DNA was assessed by qPCR and categorized as qualified (blue) or non-qualified (orange). 1 DNA sample from brushings in Preservcyt® (0.7%) and 93 DNA samples from biopsy supernatants (56%) were not analyzed (grey).

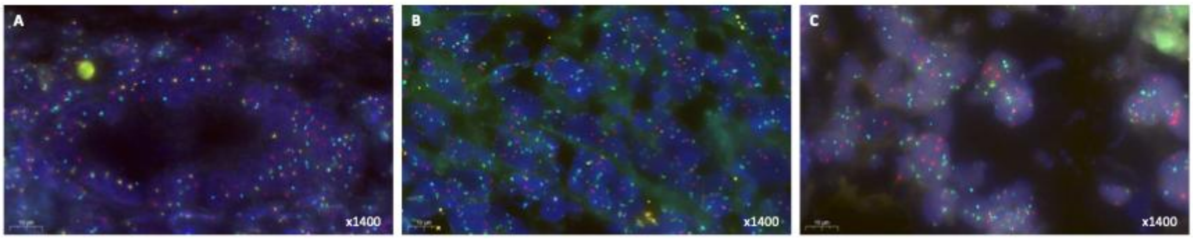

Fig. S4. Fluorescence in situ hybridization using the UroVysion® assay. (A) Procedure with normal disomic cells (two signals) for chromosomes 3 (red), 7 (green), 17 (blue), and 9p21 (yellow); (B) Procedure with polysomy; (C) Procedure with polysomy and concurrent homozygous 9p21 loss.

Table S1. Gene lists from the solid tumors panel for targeted DNA sequencing (left) and for targeted RNA sequencing (right). The 13 genes shown in bold are those for which copy number variation (CNV) analysis is feasible, as all exons or coding sequences are covered by the panel.

DNA panel		RNA panel			
ACVR1	*HRAS*	*ACTB*	*FGFR1*	*NCOA2*	*PRKD1*
AKT1	*IDH1*	*ALK*	*FGFR2*	*NF1*	*PRKD2*
ALK	*IDH2*	*ARHGAP26*	*FGFR3*	*NFIB*	*PRKD3*
BRAF	*JAK2*	*ASPSCR1*	*FN1*	*NOTCH1*	*RAF1*
BRCA1	*KIT*	*ATF1*	*FOS*	*NOTCH2*	*RELA*
BRCA2	*KRAS*	*BCOR*	*FOSB*	*NOTCH3*	*RET*
CDKN2A	*MAP2K1*	*BRAF*	*FOXO1*	*NOTCH4*	*ROS1*
CTNNB1	***MET***	*C11orf95*	*FOXR2*	*NR4A3*	*SMARCB1*
DICER1	***MYC***	*CAMTA1*	*FUS*	*NRG1*	*SRF*
DDR2	***MYCN***	*CDKN2A*	*GNAS*	*NTRK1*	*SS18*
EGFR	*MYOD1*	*CIC*	*GRM1*	*NTRK2*	*STAT6*
ERBB2	*NRAS*	*CITED1*	*HEY1*	*NTRK3*	*TEAD1*
ERBB4	***PDGFRA***	*CITED2*	*HMGA1*	*NUTM1*	*TFCP2*
FGFR1	*PIK3CA*	*CLDN18*	*HMGA2*	*OGA*	*TFE3*
FGFR2	*POLD1*	*COL1A1*	*JAZF1*	*PAX3*	*TFEB*
FGFR3	*POLE*	*CREB1*	*KIT*	*PAX7*	*TGFBR3*
FGFR4	*PRKD1*	*CREB3L1*	*MAML2*	*PBX1*	*TRIM11*
FOXL2	*PTPN11*	*CREB3L2*	*MAP3K8*	*PBX2*	*USP6*
GNA11	***PTEN***	*CREM*	*MBTD1*	*PDGFRA*	*VGLL2*
GNAQ	*RAC1*	*CSF1*	*MEIS1*	*PDGFRB*	*WT1*
GNAS	***RET***	*CTNNB1*	*MET*	*PHF1*	*YAP1*
H3F3A	***SMAD4***	*DDIT3*	*MN1*	*PLAG1*	*YWHAE*
H3F3B	***STK11***	*EGFR*	*MYB*	*PRKACA*	*ZNF444*
HIST1H3B	*TERT*	*ETV6*	*MYBL1*	*PRKACB*	
HIST1H3C	***TP53***	*EWRS1*	*NAB2*	*PRKCA*	

Table S2. Clinical, endoscopic, cytohistological, FISH and molecular data for all 127 procedures, ordered by procedure number.

PRX: procedure number X, PX: patient number X, M: male, F: female, ERCP: endoscopic retrograde cholangiopancreatography, SOC: single operator cholangioscopy.

PDF-link.

Supplementary references

1. Bournet B, Selves J, Grand D, et al. Endoscopic Ultrasound–guided Fine-Needle Aspiration Biopsy Coupled With a KRAS Mutation Assay Using Allelic Discrimination Improves the Diagnosis of Pancreatic Cancer. Journal of Clinical Gastroenterology 2015;49:50–6.
2. Syrykh C, Gorez P, Péricart S, et al. Molecular diagnosis of T-cell lymphoma: a correlative study of PCR-based T-cell clonality assessment and targeted NGS. Blood Advances 2021;5:4590–3.
3. Koeppel F, Muller E, Harlé A, et al. Standardisation of pathogenicity classification for somatic alterations in solid tumours and haematologic malignancies. European Journal of Cancer 2021;159:1–15.
4. Escudié F, Ivashchenko V, Brousset P. bialimed/SoFuR: v0.8.0 2022.
5. Bontoux C, Vigier A, Valentin T, et al. A challenging case of aggressive composite hemangioendothelioma with neuroendocrine differentiation and PTBP1::MAML2 fusion. Genes, Chromosomes and Cancer 2024;63:e2324.
